# Front-Line Therapy in EGFR Exon 19 Deletion and 21 Leu858Arg Mutations in Advanced Non-Small Cell Lung Cancer: A Network Meta-Analysis

**DOI:** 10.1155/2021/9311875

**Published:** 2021-12-13

**Authors:** Tongji Xie, Zihua Zou, Chengcheng Liu, Yixiang Zhu, Ziyi Xu, Le Wang, Junling Li, Puyuan Xing

**Affiliations:** ^1^National Cancer Center/National Clinical Research Center for Cancer/Cancer Hospital, Chinese Academy of Medical Sciences and Peking Union Medical College, Beijing 100021, China; ^2^Department of Colorectal Surgery and Oncology, Key Laboratory of Cancer Prevention and Intervention, Ministry of Education, The Second Affiliated Hospital, Zhejiang University School of Medicine, Hangzhou, China; ^3^Department of Cancer Prevention, Cancer Hospital of the University of Chinese Academy of Sciences (Zhejiang Cancer Hospital), Institute of Basic Medicine and Cancer (IBMC), Chinese Academy of Sciences, Hangzhou, Zhejiang 310022, China

## Abstract

**Objective:**

This study aimed to compare the efficacy of different first-line strategies based on different EGFR mutation types (19 deletion and 21 Leu858Arg mutations).

**Methods:**

We conducted a systematic review and network meta-analysis (NMA) by searching and analyzing RCTs on PubMed, Embase, Cochrane Library, ASCO.org, and ESMO.org, from inception to September 30^th^, 2020.

**Results:**

Nineteen RCTs involving 5450 patients were finally included in this study, covering 10 different treatment strategies. The Bayesian ranking results suggested that, in terms of PFS, in the overall population and in patients with 19del mutation, osimertinib was most likely to rank the first, with the cumulative probabilities of 41.89% and 45.73%, respectively, while for patients with 21 Leu858Arg mutation, standard of care (SoC, represents first-generation EGFR-TKIs in this NMA) + chemotherapy was most likely to rank the first, with the cumulative probabilities of 30.81% in PFS. Moreover, SoC + chemotherapy provided the best overall survival benefit for the overall population and patients with 19del, with the cumulative probabilities of 57.85% and 33.51%, respectively. In contrast, for patients with 21 Leu858Arg mutation, dacomitinib showed the most favorable overall survival, with the cumulative probabilities of 36.73%.

**Conclusions:**

In this NMA, osimertinib and SoC combined with chemotherapy would be the optimal first-line treatment options for advanced NSCLC patients harboring EGFR 19 deletion mutation and 21 Leu858Arg mutation, respectively. This finding is likely to be adopted in clinical practice and provide guidance for future clinical study design. Systematic review registration: INPLASY2020100059.

## 1. Introduction

Lung cancer leads to the highest cancer-related mortality worldwide, and non-small cell lung cancer (NSCLC) accounts for approximately 85% of overall lung cancer cases [1]. Due to the great progress in molecular diagnostic technology, several driven genes have been identified in lung adenocarcinoma, resulting in shifting the treatment for these patients from chemotherapy to targeted therapy [2]. Because numerous clinical trials demonstrated the superiority of epidermal growth factor receptor tyrosine kinase inhibitors (EGFR-TKIs) over platinum-based doublet chemotherapy in overall response rate (ORR) and progression-free survival (PFS), multiple generations of EGFR-TKIs became standard treatments in the first-line setting, including first-generation TKI gefitinib, erlotinib and icotinib, second-generation TKI dacomitinib and afatinib, and third-generation TKI osimertinib [3]. Furthermore, some synergistic combination strategies such as chemotherapy plus EGFR-TKIs or antiangiogenic drugs plus EGFR-TKIs were also investigated in some clinical trials to overcome the acquired resistance of targeted therapy [4].

What is more, the advanced NSCLC patients harboring EGFR mutation are different, including race, gender, age, smoking status, and EGFR mutation status. In terms of EGFR gene mutation, there are two common types (19 deletion mutation and 21 Leu858Arg mutation) [5] and other rare types (such as 18 Gly719Cys mutation) [6]. Data in multiple trials have shown that the efficiency of different treatment strategies might differ in various kinds of EGFR mutation statuses, especially between 19 deletion and 21 Leu858Arg mutations [7–13].

Although many previous network meta-analyses analyzed some preferable choices for EGFR-mutated advanced NSCLC based on different mutation statuses via indirect comparisons [14], some important data were still unavailable, such as final overall survival (OS) for FLAURA and NEJ026 study and PFS for CTONG-1509, RELAY, and ACTIVE study [7, 8, 15–18]. Thus, we conducted this network meta-analysis (NMA) which is widely used in the absence of head-to-head trial data [19] to further integrate the latest outcomes of randomized controlled trials and synthesize direct and indirect evidence to draw a more potent conclusion.

## 2. Method

### 2.1. Participants and Methods

This systematic review was conducted and reported under the recommendations in the Preferred Reporting Items for Systematic Reviews and Meta-Analyses (PRISMA) statement [20]. The protocol was registered on the International Platform of Registered Systematic Review and Meta-analysis Protocols (INPLASY) and is available in full at inplasy.com (https://inplasy.com/inplasy-2020-10-0059/) or in Table S1.

### 2.2. Systematic Literature Review

This systematic literature review was conducted to identify clinical trials assessing the efficacy of EGFR-TKIs, including PFS and OS. The PubMed, Embase, and Cochrane Central Register of Controlled Trials databases were searched from inception to September 30^th^, 2020. Under the guidelines of PICOs, combinations of MeSH terms and keywords related to “NSCLC,” “EGFR,” “TKI,” “PFS,” “OS,” and “Randomized Controlled Trial (RCT)” were applied in the literature search. Besides, literature was further supplemented in conferences of the American Society of Clinical Oncology, European Society of Medical Oncology, European Cancer Conference, and World Conference on Lung Cancer. Detailed search strategies are available in Table S2.

### 2.3. Outcome Definition

The primary outcome was PFS, defined as the time from randomization to the first documented disease progression or death from any cause. The secondary outcome was OS, which was defined as the time from date of randomization to death from any cause.

### 2.4. Selection of Studies for the NMA

Eligible studies need to meet all the following inclusion criteria: (1) study population: patients with advanced NSCLC harboring EGFR mutation; (2) interventions: EGFR-TKIs with or without antivascular endothelial growth factor (VEGF); (3) comparators: EGFR-TKIs or chemotherapy; (4) outcomes: OS, PFS; and (5) study design: RCTs.

Studies were excluded if they met any of the following exclusion criteria: (1) no EGFR mutation patients; (2) no intervention of EGFR-TKIs; (3) EGFR-TKIs not as first-line treatment; (4) no survival data; (5) duplicated studies; and (6) review, comment, editor opinion, or protocol.

### 2.5. Data Extraction and Risk of Bias Assessment

According to predefined eligibility criteria by the research working group, the titles and abstracts of all identified records were initially screened. Then, potentially eligible studies were assessed by full text. For the final included studies, data extraction and risk of assessment were further performed. The above contents were conducted by Tongji Xie and Zihua Zou independently, and a third expert (Puyuan Xing) was invited to arbitrate until reaching a consensus in case of any disagreement.

A proform designed by the review working group was done for data extraction, including the following information: (1) basic information: name of the study, year of publication, country; (2) trials design: design type, patient characteristics, sample size, therapies in intervention, and control group; and (3) outcomes: data on PFS and OS.

The Cochrane risk-of-bias tool [21] was used to assess random sequence generation (selection bias), allocation concealment (selection bias), blinding of participants and personnel (performance bias), blinding of outcome assessment (detection bias), incomplete outcome data (attrition bias), selective reporting (reporting bias), and other biases.

### 2.6. Network Meta-Analyses

For both survival outcomes, the natural log hazard ratio (HR) versus the reference arm and its associated standard error (SE) were used as inputs for the NMAs. The network plots were drawn using Stata software (version 15.0) to show the interaction of different treatment regimens in the included studies [22]. Heterogeneity across included studies was assessed by Q test and *I*^2^. Heterogeneity was considered low, moderate, or high for estimated *I*^2^ values under 25%, between 25% and 50%, and over 50%, respectively [23].

Bayesian network meta-analysis was applied due to its advantages of accommodating complex situations (accounting for the effect of study-specific covariates, resulting in exact estimates in the presence of limited information) and providing a more straightforward method for conducting probabilistic statements and predictions on the treatment effects. The Bayesian framework was performed by a Markov Chain Monte Carlo simulation technique in R software (version 3.6.3). We utilized the “gemtc” and “rjags” packages and chose the random effect model, using the odds ratio (OR) as the effect quantity and 95% credible interval (CI) to compare the intervention measures [24].

Based on the previous experience and published literature, we chose the fixed model in the registration for this NMA. When we conducted the analysis, most NMAs showed low heterogeneities because most evaluated interventions were from only one or two studies with almost identical population characteristics. However, for PFS in all patients, high heterogeneities could be found in the comparisons between Chemotherapy and Afatinib (*I*^2^ = 75.7%), standard of care (SoC, represents first-generation EGFR-TKIs in this NMA), and chemotherapy (*I*^2^ = 74.8%). For PFS in patients with 19del mutation, comparisons of SoC and chemotherapy (*I*^2^ = 70.9%) showed high heterogeneities. In addition, for PFS in patients with 21 Leu858Arg mutation, comparisons of chemotherapy and afatinib (*I*^2^ = 65.6%), SoC and chemotherapy (*I*^2^ = 55.2%), and SoC + chemotherapy and SoC (*I*^2^ = 53.7%) showed high heterogeneities. For OS in all patients, high heterogeneities could be found in the comparisons between chemotherapy and afatinib (*I*^2^ = 70.7%), and SoC and osimertinib (*I*^2^ = 65.4%). For OS in patients with 19del mutation, comparisons of SoC and Afatinib (*I*^2^ = 64.1%), and SoC and chemotherapy (*I*^2^ = 54.6%) also showed high heterogeneities (data were not shown). Finally, given the high heterogeneities mentioned above, the random effects consistency model was used to guarantee the model's robustness.

There are four different sets of initial values to fit the model. For both the PFS and OS, 10,000 sample iterations were generated with 5 000 burn-ins and a thinning interval of 5. We evaluated convergence of iterations by visual inspection of the four chains to establish homogenous parameter estimates in accordance with the density plot (Figure S1) [25]. Under the Bayesian framework, NMA estimated the overall rankings of treatments based on the surface under the cumulative ranking curve for each, which equals 1 when a treatment is certain to be the best and 0 when a treatment is certain to be the worst.

More importantly, two key assumptions in support of the NMA are transitivity (the exchangeability across included studies to compare two treatments via a third one) and consistency (that the direct and indirect estimates are statistically similar). To guarantee the transitivity, we identified randomized controlled trials with strict patient allocation and optimized the same condition for all evaluated treatments. Inconsistency was evaluated by comparing the fit of consistency and inconsistency models [26]. If a direct comparison existed simultaneously between the 2 interventions, a node splitting technique was used to evaluate the network consistency by determining the difference between the indirect and direct estimates.

## 3. Results

### 3.1. Characteristics of Included Studies

**Figure 1:**
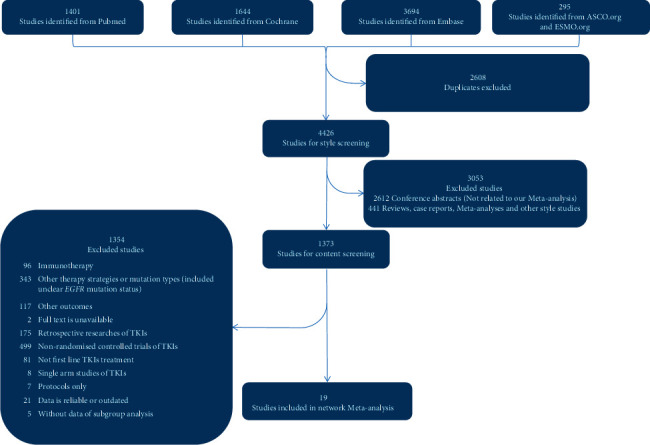
Flowchart depicting the process of identifying the studies. ASCO : American Society of Clinical Oncology; ESMO : European Society for Medical Oncology; EGFR: epidermal growth factor receptor; TKIs: tyrosine kinase inhibitors.

A total of 19 randomized controlled trials were finally included in this study, covering 10 different treatments. Detailed information for inclusion and exclusion is shown in Figure 1.

Table 1 summarizes studies included in this meta-analysis [7–13, 15–18, 27–45]. The range of median PFS follow-up time was between 2.7 and 45.0 months. The range of median OS follow-up time was between 27.0 and 59.1 months. The mean sample size was 287 patients (range, 81 to 556 patients).

### 3.2. Quality of Reporting Evidence

The quality assessment for included studies was mainly based on the Cochrane handbook. The overall quality is of low-to-medium bias risk.

**Figure 2:**
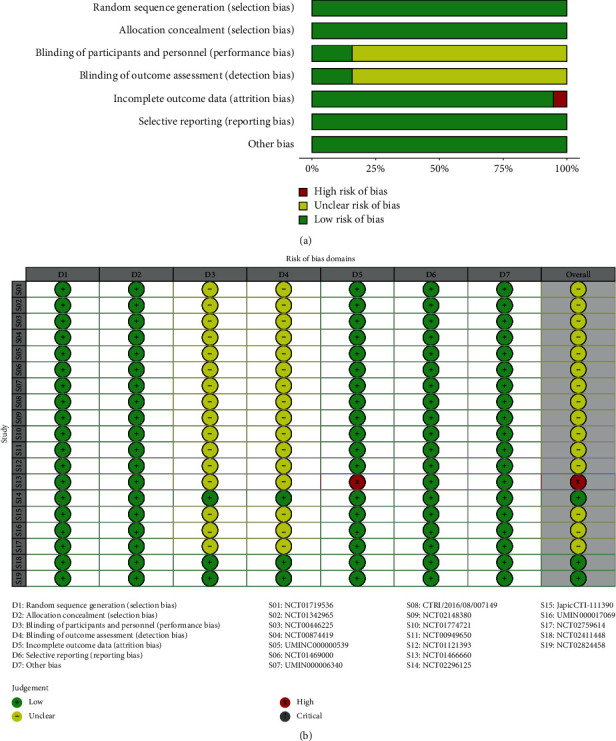
Risk of bias assessment. (a) Risk of bias assessment: overall risk of bias for all included trials. (b) Risk of bias summary: overall risk of bias for all included trials.

All included studies described the method used to generate the allocation sequence, conceal the allocation sequence, and report the related outcomes based on the trial's protocol. No serious census data could be found in most studies, except for the trial NCT01466660 [27, 28] with high incomplete outcome bias: 34 patients recruited in this trial did not have their ethnic origin recorded [27]. However, these 34 patients were sorted into a non-Asian group when the OS of the trial was reported [28]. Although we do not analyze the ethnic information of patients, the bias of the trial NCT01466660 [27, 28] adversely affects the reliability of its results. More importantly, most studies did not state the method for blinding in both the intervention and outcome. Details for the quality evaluation of the included literature are listed in Figure 2.

### 3.3. Network Meta-Analysis

**Figure 3:**
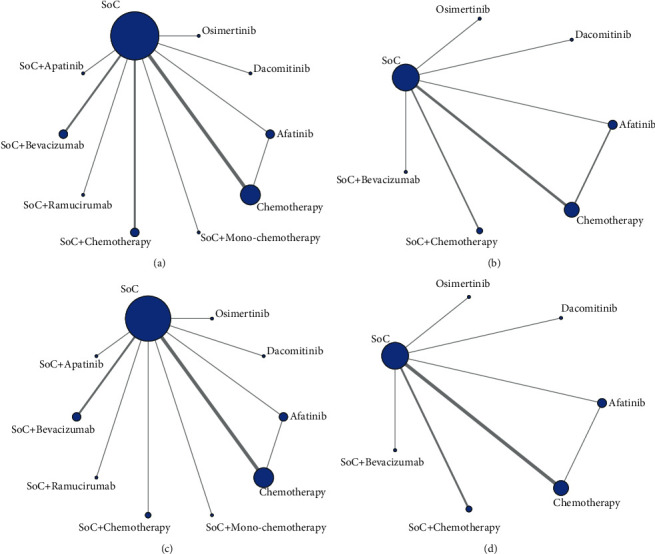
Network diagrams of comparisons on different outcomes of treatments in different EGFR (epidermal growth factor receptor) mutation type groups of patients with non-small cell lung cancer (NSCLC). (a) PFS among patients with 19 del mutation; (b) OS among patients with 19 del mutation; (c) PFS among patients with 21L858R mutation; (d) OS among patients with 21L858R mutation. PFS: progression-free survival; OS: overall survival; SoC: standard of care, representing first-generation EGFR-TKIs in this network meta-analysis (including gefitinib, erlotinib, and icotinib).

Network meta-analysis included all treatments for PFS, while there were 16 studies with the outcome of OS. For patients with 19del mutation, all identified literature reported PFS (Figure 3(a)), while 11 had the outcome of OS (Figure 3(b)). For patients with 21 Leu858Arg mutation, 18 included studies reported PFS (Figure 3(c)), while 11 studies reported the outcome of OS (Figure 3(d)).

### 3.4. Progression-Free Survival: Overall and Results Specific to Mutation Type

**Figure 4:**
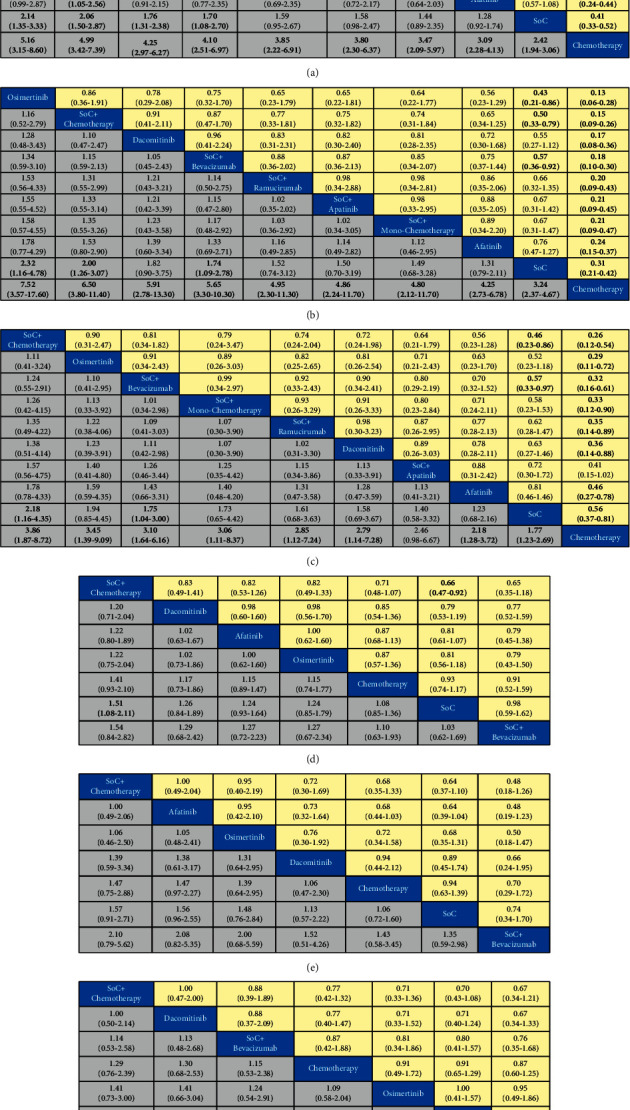
Pooled estimates of the network meta-analysis. Pooled odds ratios (95% credible intervals). Data in each cell are hazard or odds ratios (95% credible intervals) for the comparison of row-defining treatment versus column-defining treatment. Hazard ratios less than 1 and odds ratios more than 1 favor row-defining treatment. Significant results are in bold. PFS: progression-free survival; OS: overall survival; SoC: standard of care, representing first-generation EGFR-TKIs in this network meta-analysis (including gefitinib, erlotinib, and icotinib). (a) PFS in all patients; (b) PFS in patients with 19del mutation; (c) PFS in patients with 21L858R mutation; (d) OS in all patients; (e) OS in patients with 19del mutation; (f) OS in patients with 21L858R mutation.

For PFS for all patients (Figure 4(a)), chemotherapy had lowest efficacy, when compared with osimertinib (HR 0.19, 95% CI 0.12–0.32), SoC + chemotherapy (HR 0.20, 95% CI 0.14–0.29), SoC + bevacizumab (HR 0.24, 95% CI 0.16–0.34), dacomitinib (HR 0.24, 95% CI 0.14–0.40), SoC + monochemotherapy (HR 0.26, 95% CI 0.15–0.45), SoC + ramucirumab (HR 0.26, 95% CI 0.16–0.44), SoC + apatinib (HR 0.29, 95% CI 0.17–0.48), afatinib (HR 0.32, 95% CI 0.24–0.44), and SoC (HR 0.41, 95% CI 0.33–0.52). In addition, compared with osimertinib (HR 0.47, 95% CI 0.30–0.74), SoC + chemotherapy (HR 0.49, 95%CI 0.35–0.67), SoC + bevacizumab (HR 0.57, 95% CI 0.42–0.77), and dacomitinib (HR 0.59, 95% CI 0.37–0.93), SoC had lower efficacy. What is more, afatinib (HR 0.62, 95% CI 0.39–0.96) was inferior to SoC + chemotherapy. As for other comparisons, no significant difference could be found.

In terms of PFS for patients with 19del mutation (Figure 4(b)), compared with chemotherapy, osimertinib (HR 0.13, 95% CI 0.06–0.28), SoC + chemotherapy (HR 0.15, 95% CI 0.09–0.26), dacomitinib (HR 0.17, 95% CI 0.08–0.36), SoC + bevacizumab (HR 0.18, 95% CI 0.10–0.30), SoC + ramucirumab (HR 0.20, 95% CI 0.09–0.43), SoC + apatinib (HR 0.21, 95% CI 0.09–0.45), SoC + monochemotherapy (HR 0.21, 95% CI 0.09–0.47), afatinib (HR 0.24, 95% CI 0.15–0.37), and SoC (HR 0.31, 95% CI 0.21–0.42) showed superior efficacy. Additionally, compared with osimertinib (HR 0.43, 95% CI 0.21–0.86), SoC + chemotherapy (HR 0.50, 95% CI 0.33–0.79), and SoC + bevacizumab (HR 0.57, 95% CI 0.36–0.92), SoC had poorer PFS. For other interventions, no distinguished differences could be found.

For PFS in patients with 21 Leu858Arg mutation (Figure 4(c)), in comparison with SoC, SoC + chemotherapy (HR 0.46, 95% CI 0.23–0.86) and SoC + bevacizumab (HR 0.57, 95% CI 0.33–0.97) demonstrated more favorable prognosis. Particularly, chemotherapy showed the worst results in PFS, compared with SoC + chemotherapy (HR 0.26, 95% CI 0.12–0.54), osimertinib (HR 0.29, 95% CI 0.11–0.72), SoC + bevacizumab (HR 0.32, 95% CI 0.16–0.61), SoC + monochemotherapy (HR 0.33, 95% CI 0.12–0.90), SoC + ramucirumab (HR 0.35, 95% CI 0.14–0.89), dacomitinib (HR 0.36, 95% CI 0.14–0.88), afatinib (HR 0.46, 95% CI 0.27–0.78), and SoC (HR 0.56, 95% CI 0.37–0.81). From the available comparisons, no other significant differences could be found.

### 3.5. Overall Survival: Overall and Results Specific to Mutation Type

For OS in all patients (Figure 4(d)), SoC had lower efficacy than SoC + chemotherapy (HR 0.66, 95% CI 0.47–0.92). No superior treatment could be found among SoC + chemotherapy, dacomitinib, afatinib, osimertinib, chemotherapy, SoC, and SoC + bevacizumab for patients with mutations of 19del (Figure 4(e)) or 21 Leu858Arg (Figure 4(f)).

### 3.6. Rank Probabilities

**Figure 5:**
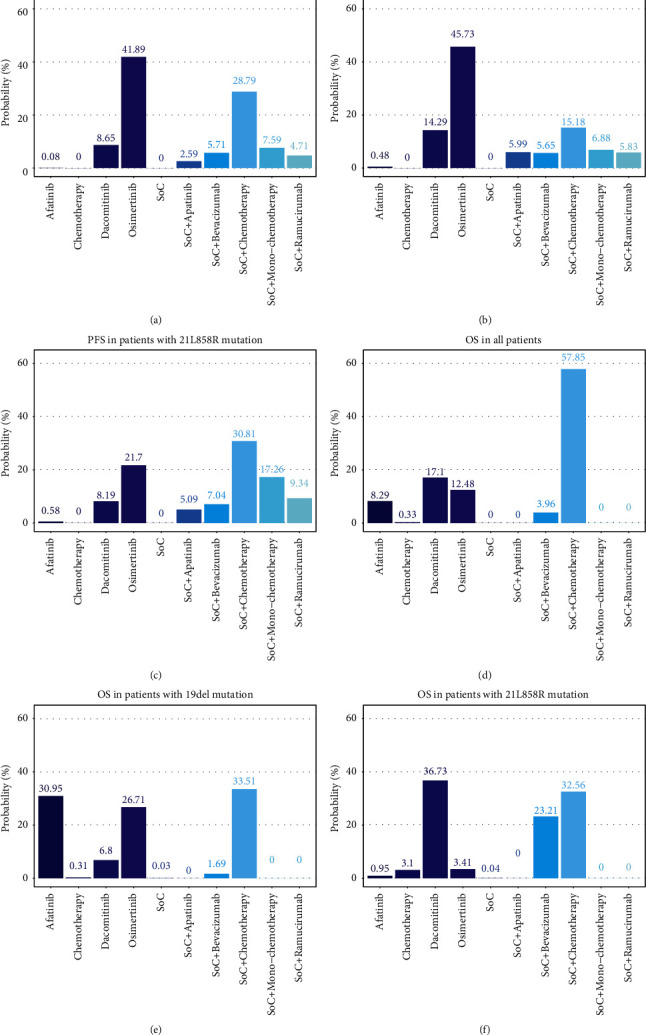
Surface under the cumulative ranking curve values for progression-free survival and overall survival. SUCRA values showing the percentage of efficacy for each treatment to be ranked the first compared with an imaginary treatment that will be ranked first without uncertainty. For each outcome, treatment with the highest SUCRA value is ranked the most efficacious. SUCRA: surface under the cumulative ranking curve; PFS: progression-free survival; OS: overall survival; SoC: standard of care, representing first-generation EGFR-TKIs in this network meta-analysis (including gefitinib, erlotinib, and icotinib). (a) PFS in all patients; (b) PFS in patients with 19del mutation; (c) PFS in patients with 21L858R mutation; (d) OS in all patients; (e) OS in patients with 19del mutation; (f) OS in patients with 21L858R mutation.

Bayesian ranking profiles of evaluated treatments in different populations could be found in Figure S2. In general, the Bayesian ranking results were almost in line with the pooled analysis using HR. In terms of PFS, in the overall population (Figure 5(a)) and patients with 19del mutation (Figure 5(b)), osimertinib was most likely to rank first, with the cumulative probabilities of 41.89% and 45.73%, respectively. Nonetheless, for patients with 21 Leu858Arg mutation (Figure 5(c)), SoC + chemotherapy demonstrated the most favorable PFS (30.81%). For OS, SoC + chemotherapy ranked at the top of the list for the overall population (Figure 5(d)) and patients with 19del mutation (Figure 5(e)), with the cumulative probabilities of 57.85% and 33.51%, respectively. For patients with 21 Leu858Arg mutation (Figure 5(f)), dacomitinib provided the best OS (36.73%).

### 3.7. Inconsistency Assessment

The fit of the consistency model was similar to or better than that of the inconsistency model (Table S3). No significant differences in comparisons could be found between direct and indirect estimates in the node splitting analysis (*P* > 0.05) (Table S4).

## 4. Discussion

Compared with previous studies, our NMA showed the following advantages. Firstly, our study ensured the homogeneity of the study population. Because some patients harboring uncommon EGFR mutations were included in some clinical trials, such as Lux-Lung-3 [43, 44] and Lux-Lung-6 [44, 45], we ruled out this part of patients and only included patients with common EGFR mutations in the final analysis. Secondly, data extracted from some RCTs served as a bridge to systematically explore the optimal treatment drugs among six commonly used EGFR-TKIs and three antivascular agents for patients with 19 deletion and 21 Leu858Arg mutations, respectively. Thirdly, although many previous NMAs analyzed some preferable choices for EGFR-mutated advanced NSCLC based on different mutation status [14], some important data were not available at that time (these include final OS for FLAURA and NEJ026 study, PFS for CTONG-1509, RELAY, and ACTIVE study) [7, 8, 15–18]. We conducted this NMA by further integrating the latest information of RCTs in order to draw a more compelling conclusion. Fourth, our NMA systematically compared multiple therapeutic strategies, including three different anti-VEGF agents, bevacizumab [12, 13, 15, 16, 29], ramucirumab [17], and apatinib [18], not available in other NMAs.

In NMA, we comprehensively compared the efficacy of multiple first-line treatments, including all available EGFR-TKIs, cytotoxic agents, and combination strategies for advanced NSCLC patients harboring two different common EGFR mutations. The results suggested that osimertinib was considered the optimal treatment strategy for all patients and patients with EGFR exon 19 deletion in providing the best PFS. First-generation EGFR-TKIs plus chemotherapy was regarded as the best treatment strategy for patients with EGFR exon 21 Leu858Arg mutation with the best PFS. Combination treatment of first-generation EGFR-TKIs with chemotherapy showed the best efficiency in terms of OS for all patients and patients harboring EGFR exon 19 deletion. AS for the best choice for patients with EGFR exon 21 Leu858Arg mutation in providing the best OS, two methods employed by our NMA provided slightly different conclusions: in pooled analysis (Figure 4(f)), first-generation EGFR-TKIs plus chemotherapy was the best choice, but in Bayesian ranking profiles (Figure 5(f)), dacomitinib was considered the best. Given that the HR/OR for SoC + chemotherapy versus dacomitinib was equal to 1 and the cumulative probabilities of dacomitinib (36.73%) and SoC + chemotherapy (32.56%) to rank the first were close, we deduced that dacomitinib and SoC + chemotherapy could demonstrate similar performance in prolonging OS for patients with EGFR exon 21 Leu858Arg mutation. Combination treatments of first-generation EGFR-TKIs with different anti-VEGF agents show the identical tendency in providing better PFS for all patients (Figure 4(a)) and patients harboring different common mutations (Figures 4(b) and 4(c)) compared to SoC and chemotherapy: first-generation EGFR-TKIs with bevacizumab was the best, followed by ramucirumab and apatinib.

The heterogeneity and inconsistency assessment suggested that minor heterogeneities could be found in most comparisons from one or two studies. The quality assessment for included studies showed low-to-medium bias risk. On the whole, the Bayesian ranking results were almost in line with the pooled analysis using hazard and odds ratios. Therefore, the results we got could be considered robust.

Osimertinib can provide long PFS and translate PFS benefit to the improvement in OS due to its irreversible inhibition of EGFR and additional inhibition of EGFR exon 20 Thr790Met mutated type EGFR, which accounts for about 50–60% acquired resistance of first- and second-generation EGFR-TKIs [46, 47]. The high efficiency of first-generation EGFR-TKIs plus chemotherapy might be attributed to this combination strategy which could effectively cope with drug resistance. On the one hand, pemetrexed plus gefitinib could prevent the resistance of gefitinib in EGFR 19del mutated NSCLC cell lines by avoiding the occurrence of the EGFR exon 20 Thr790Met mutation or epithelial to mesenchymal transition [48]. On the other hand, the combination of erlotinib or gefitinib with pemetrexed can prolong the benefits of patients with acquired resistance to erlotinib or gefitinib [49]. Furthermore, gefitinib could reverse the chemotherapy resistance in the NSCLC cell line [50]. Therefore, whether osimertinib or the combination of the first-line EGFR-TKIs with chemotherapy can be considered the optimized treatment strategy for advanced EGFR mutated NSCLC patients.

As a secondary endpoint in most studies, OS is impacted by multiple factors, and thus we mainly analyzed the difference of PFS in this NMA. Different statuses of EGFR mutation might influence the choice of treatment strategies for advanced EGFR mutated NSCLC patients in acquiring the best PFS. Some studies imply that EGFR-TKIs are more efficient in patients with 19del mutation than with 21 Leu858Arg mutation [51, 52]. A possible explanation might be that the EGFR exon 21 Leu858Arg mutation is accompanied by a more frequent appearance of EGFR exon 20 Thr790Met mutations [53, 54] and concomitant mutations (such as *TP53*, *PIK3CA*, BRAF) [55], which are associated with higher resistance and worse response of EGFR-TKIs [56–59], compared with EGFR exon 19del mutation [59]. Therefore, the third-generation EGFR-TKI osimertinib should be more efficient in patients harboring 19del mutation because of its irreversible inhibition of EGFR, which is consistent with our results (pooled odds ratio of osimertinib versus SoC + chemotherapy is 0.86, 0.36–1.91 in Figure 4(b), and in Figure 5(b), osimertinib was most likely to rank the first in 19del mutated patients). First-generation EGFR-TKIs plus chemotherapy should be more efficient in 21 Leu858Arg mutated patients who are liable to show drug resistance due to its effectively preventing patients from multimechanism resistance to first-generation EGFR-TKIs [48], which is also in accordance with our results (pooled odds ratio of SoC + chemotherapy versus osimertinib is 0.90, 0.31–2.47 in Figure 4(c), and in Figure 5(c), SoC + chemotherapy was most likely to rank the first in 21 Leu858Arg mutated patients). Additionally, the optimized choice for all patients in providing longer PFS is osimertinib, which might owe to the amount of 19del mutated patients is larger than 21 Leu858Arg mutated patients in most selected RCTs (pooled odds ratio of osimertinib versus SoC + chemotherapy is 0.96, 0.55–1.68 in Figure 4(a), and in Figure 5(a), osimertinib was most likely to rank the first in all patients).

In terms of OS, the first-generation EGFR-TKIs plus chemotherapy shows the best efficiency for all patients and patients harboring different mutation types, which might be associated with the activation of chemotherapy to the immune system (eliminating immunosuppression cells [60, 61], generating memory T cells [62], etc.) and activated immune system.

Our results were similar to previous findings. In terms of PFS, Zhao et al. found that osimertinib and gefitinib plus pemetrexed based chemotherapy were considered the best treatment options for 19del mutated patients and 21 Leu858Arg mutated patients, respectively, which was in consistent with our results [14]. As for OS, Zhao et al. considered afatinib and dacomitinib were the most favorable treatments for 19del mutated patients and 21 Leu858Arg mutated patients respectively [14]. Zhao et al.'s result of OS was slightly different from ours' which could be attributed to immature subgroup results of NEJ009 study at that time [32]. In Alanazi et al.'s NMA, osimertinib and dacomitinib ranked first in all patients in terms of PFS and OS respectively, which was incongruent with our findings [63]. It should be noted that combination treatment strategies were not included in their research.

We have also found that the combination treatment of first-generation EGFR-TKIs with different anti-VEGF agents show the identical tendency in providing better PFS for all patients and patients harboring different common mutations: first-generation EGFR-TKIs with bevacizumab is the best, with ramucirumab is the next and with apatinib is the worst. The reasons for this tendency might be as follows: (1) all selected RCTs about first-generation EGFR-TKIs plus bevacizumab [12, 13, 15, 16, 29] are open-label, while the RCTs about the combination with ramucirumab [17] or apatinib [18] are double-blind, which might lead to overestimating the benefits of bevacizumab; (2) the molecular structure of these anti-VEGF agents and their targets are different. Bevacizumab is a VEGF targeted IgG1 monoclonal antibody; ramucirumab is a VEGFR targeted IgG1 monoclonal antibody; apatinib is a VEGFR-TKIs, which might have some influences on their efficacy (monoclonal antibody could mediate antibody-dependent cell-mediated cytotoxicity and opsonization, which could increase the apoptosis of vascular endothelial and the elimination of VEGF, respectively).

There are still some limitations in this study. Firstly, this NMA uses some data from the experimental group or control group in some studies as a “bridge” to indirectly compare the advantages and disadvantages of different treatment strategies. However, the most direct evidence should come from a direct comparison of two or more agents, and the indirect comparison results may be distorted. Secondly, although our NMA suggests first-generation EGFR-TKIs combined with chemotherapy are the optimal first-line treatment option for advanced NSCLC patients harboring EGFR 21 Leu858Arg mutation, there is no RCT comparing the second- or third-generation EGFR-TKIs plus chemotherapy with chemotherapy or other treatments, and whether chemotherapy combining the second- or third-generation EGFR-TKIs would be more effective than the first-generation, EGFR-TKI should be further investigated. Thirdly, heterogeneity might be influenced by the complexity of subsequent treatment options in different trials when OS was considered the endpoint for evaluating the efficacy of various treatment strategies. Therefore, PFS was taken as the primary endpoint in this meta-analysis. Finally, NSCLC patients, with other driver gene changes or EGFR uncommon mutations, or patients of other kinds of cancer with gene mutations also have the problem of “optimal treatment,” which needs to be further studied by clinical workers and researchers.

## 5. Conclusions

This NMA suggests that osimertinib and first-generation EGFR-TKIs combined with chemotherapy would be the optimal first-line treatment option for advanced NSCLC patients harboring EGFR 19 deletion mutation and 21 Leu858Arg mutation, respectively.

## Figures and Tables

**Table 1:** Baseline characteristics of studies included in the network meta-analysis of patients with advanced epidermal growth factor receptor (EGFR) mutated non-small cell lung cancer.

Study	Region	Year (enrollment of patients)	Phase	Clinical trial number	Sample size (no)	Median age	Female (%)	Adenocarcinoma (%)	BM status	Ethnicity	Nonsmoking (%)	EGFR mutation type		Intervention Arm	Control Arm	Overcomes	
												Exon 19 deletion	Leu858Arg			PFS	OS
FLAURA [7–9]	Global (multicenter)	2014–2016	III (double-blind)	NCT02296125	279/277	64/64	63.8/62.1	99/98	BM and non-BM	Asian and non-Asian	65.2/63.2	175/174	104/103	Osimertinib	SoC	Yes	Yes
ARCHER1050 [10, 11]	Global (multicenter)	2013	III (open-label)	NCT01774721	227/225	62/61	64.3/55.6	NA	Non-BM	Asian and non-Asian	64.8/64.0	134/133	93/92	Dacomitinib	SoC	Yes	Yes
LUX-LUNG-7 [27, 28]	Global (multicenter)	2011	IIb (open-label)	NCT01466660	160/159	63/63	56.9/66.7	99/99	BM and non-BM	Asian and non-Asian	66.3/66.7	93/93	67/66	Afatinib	SoC	Yes	Yes
JO25567 [12, 13]	Japan (multicenter)	2011–2012	II (open-label)	JapicCTI-111390	75/77	67/67	60.0/66.2	99/99	Non-BM	Asian	56.0/58.4	40/40	35/37	SoC + bevacizumab	SoC	Yes	Yes
NEJ026 [15, 29]	Japan (multicenter)	2015–2016	III (open-label)	UMIN000017069	112/112	67/68	63.4/65.2	98/100	BM and non-BM	Asian	58.0/57.1	56/55	56/57	SoC + bevacizumab	SoC	Yes	Yes
CTONG-1509 [16]	China (multicenter)	2016–2017	III (open-label)	NCT02759614	157/154	57/59	61.8/62.3	100/100	BM and non-BM	Asian	NA	82/79	75/75	SoC + bevacizumab	SoC	Yes	No
RELAY [17]	Global (multicenter)	2016–2018	III (double-blind)	NCT02411448	224/225	65/64	62.9/63.1	96/97	Non-BM	Asian and non-Asian	59.8/61.8	123/120	99/105	SoC + ramucirumab	SoC	Yes	No
ACTIVE [18]	China (multicenter)	NA	III (double-blind)	NCT02824458	157/156	57/60	58.0/60.3	NA	BM and non-BM	Asian	73.2/77.6	81/83	74/73	SoC + apatinib	SoC	Yes	No
JMIT [30, 31]	East-Asia (multicenter)	2012	II (open-label)	NCT01469000	126/65	62/62	65.1/63.1	NA	NA	Asian	64.3/72.3	65/40	52/23	SoC + chemotherapy	SoC	Yes	Yes
NEJ009 [32]	Japan (multicenter)	2011	III (open-label)	UMIN000006340	170/172	64.8/64.0^*∗*^	67.1/62.8	99/99	BM and non-BM	Asian	56.5/56.4	93/95	69/67	SoC + chemotherapy	SoC	Yes	Yes
Noronha. et al. [33]	India (monocenter)	2016–2018	III (open-label)	CTRI/2016/08/007149	174/176	54/56	49.4/47.2	98/97	BM and non-BM	Asian	83.3/85.2	107/109	NA	SoC + chemotherapy	SoC	Yes	Yes
Han.et al [34, 35]	China (monocenter)	2011	II (open-label)	NCT02148380	40/41	NA	62.5/56.1	100/100	BM and non-BM	Asian	67.5/65.9	21/21	19/20	SoC + chemotherapy	SoC	Yes	Yes
CONVINCE [36]	China (multicenter)	2013–2014	III (open-label)	NCT01719536	148/137	56/56	70.9/69.3	100/100	BM and non-BM	Asian	78.4/78.8	80/74	68/63	SoC	Chemotherapy	Yes	Yes
ENSURE [37]	China (multicenter)	2011–2012	III (open-label)	NCT01342965	110/107	57.5/56	61.8/60.7	95/94	Non-BM	Asian	71.8/69.2	58/61	52/46	SoC	Chemotherapy	Yes	Yes
EURTAC [38]	Europe (multicenter)	2007–2011	III (open-label)	NCT00446225	86/87	65/65	67.4/78.2	95/90	BM and non-BM	Non-Asian	66.3/72.4	57/58	29/29	SoC	Chemotherapy	Yes	Yes
OPTIMAL [39, 40]	China (multicenter)	2008–2009	III (open-label)	NCT00874419	82/72	57/59	58.5/59.7	88/86	NA	Asian	72.0/69.4	43/39	39/33	SoC	Chemotherapy	Yes	Yes
WJTOG3405 [41, 42]	Japan (multicenter)	2006–2009	III (open-label)	UMIN000000539	86/86	64/64	68.6/69.8	97/98	NA	Asian	70.9/66.3	50/37	36/49	SoC	Chemotherapy	Yes	Yes
LUX-LUNG-3 [43, 44]	Global (multicenter)	2009	III (open-label)	NCT00949650	230/115	61.5/61	63.9/67.0	100/100	BM and non-BM	Asian and non-Asian	67.4/70.4	113/57	91/47	Afatinib	Chemotherapy	Yes	Yes
LUX-LUNG-6 [44, 45]	East-Asia (multicenter)	2010	III (open-label)	NCT01121393	242/122	58/58	64.0/68.0	100/100	BM and non-BM	Asian	74.8/81.1	124/62	92/46	Afatinib	Chemotherapy	Yes	Yes

Data are expressed as intervention/control unless indicated otherwise. NA: not available; BM: brain metastases; SoC: standard of care, representing first-generation epidermal growth factor receptor tyrosine kinase inhibitors in this network meta-analysis (including gefitinib, erlotinib, and icotinib). ^*∗*^Median age is not given, so the mean age is shown here.

## Data Availability

The data were extracted from published articles or abstracts.

## Supplementary Materials

Supplementary Materials There are 4 supplementary tables and 2 supplementary figures for this paper.
