# Supplementary material for: Front-Line Therapy in EGFR Exon 19 Deletion and 21 Leu858Arg Mutations in Advanced Non-Small Cell Lung Cancer: A Network Meta-Analysis
Source: Evid Based Complement Alternat Med. 2021 Dec 13;2021:9311875. doi: 10.1155/2021/9311875 (PMC8687779; doi:10.1155/2021/9311875)
Supplement: Supplementary Materials — There are 4 supplementary tables and 2 supplementary figures for this paper. [file 9311875.f1.zip › 9311875.f1/FigureS1 (1).pdf]

Figure S1 Trace plot, Density plot and convergence diagnostics plot for Network Meta Analysis

The number from 1 to 10 in the title of trace plot, density plot and convergence diagnostics plot represents ten different treatments: 1 represents Afatinib; 2 represents Chemotherapy; 3 represents Dacomitinib; 4 represents Osimertinib; 5 represents SoC; 6 represents SoC+Apatinib; 7 represents SoC+Bevacizumab; 8 represents SoC+Chemotherapy; 9 represents SoC+Mono−chemotherapy; 10 represents SoC+Ramucirumab. PFS: progression free survival; OS: overall survival.

a. PFS in all patients; b. PFS in patients with 19 del mutation; c. PFS in patients with 21L858R mutation;
d. OS in all patients; e. OS in patients with 19 del mutation; f. OS in patients with 21L858R mutation

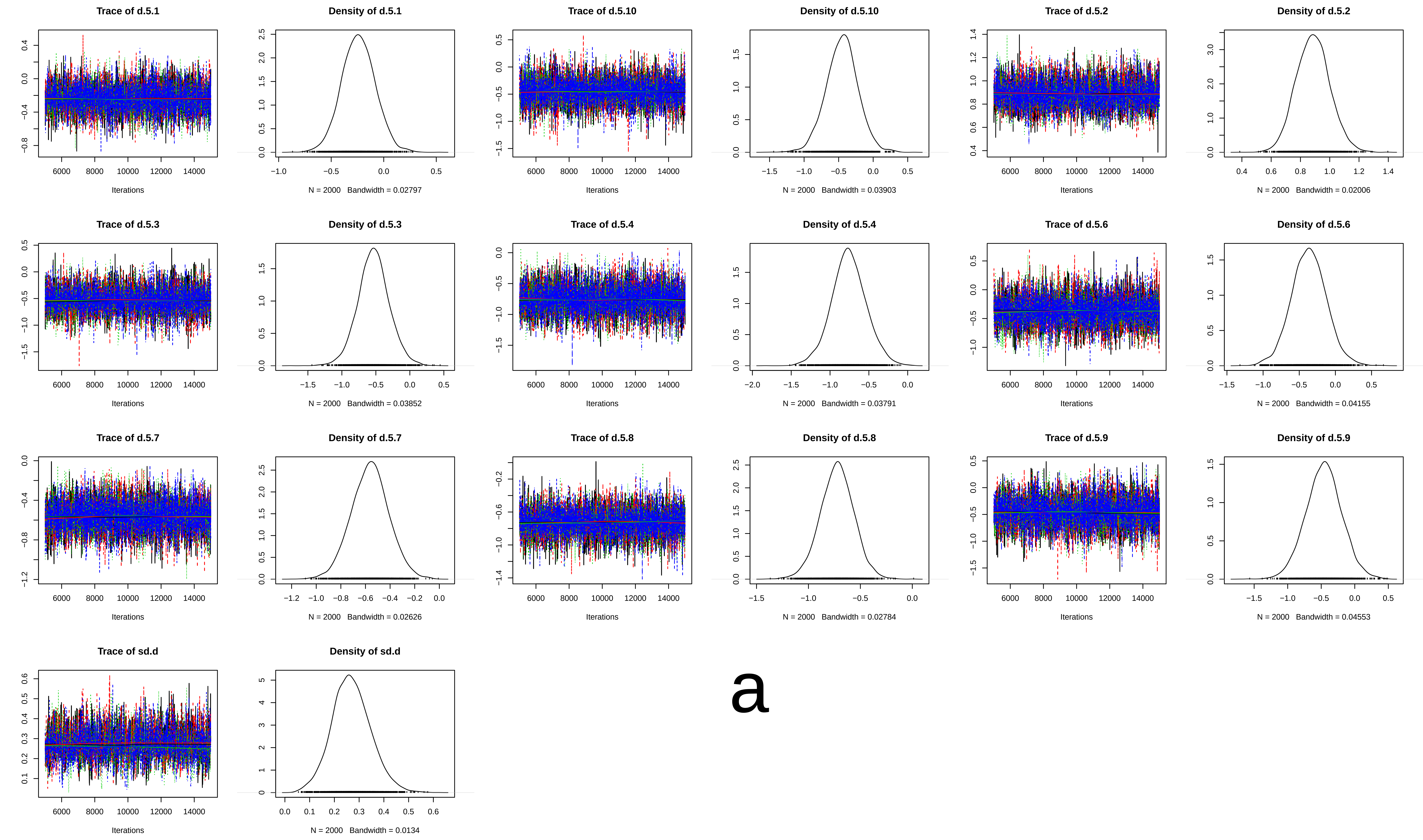

Trace of d.5.1
Iterations
Density of d.5.1
N = 2000 Bandwidth = 0.02797
Trace of d.5.10
Iterations
Density of d.5.10
N = 2000 Bandwidth = 0.03903
Trace of d.5.2
Iterations
Density of d.5.2
N = 2000 Bandwidth = 0.02006
Trace of d.5.3
Iterations
Density of d.5.3
N = 2000 Bandwidth = 0.03852
Trace of d.5.4
Iterations
Density of d.5.4
N = 2000 Bandwidth = 0.03791
Trace of d.5.6
Iterations
Density of d.5.6
N = 2000 Bandwidth = 0.04155
Trace of d.5.7
Iterations
Density of d.5.7
N = 2000 Bandwidth = 0.02626
Trace of d.5.8
Iterations
Density of d.5.8
N = 2000 Bandwidth = 0.02784
Trace of d.5.9
Iterations
Density of d.5.9
N = 2000 Bandwidth = 0.04553
Trace of sd.d
Iterations
Density of sd.d
N = 2000 Bandwidth = 0.0134
a

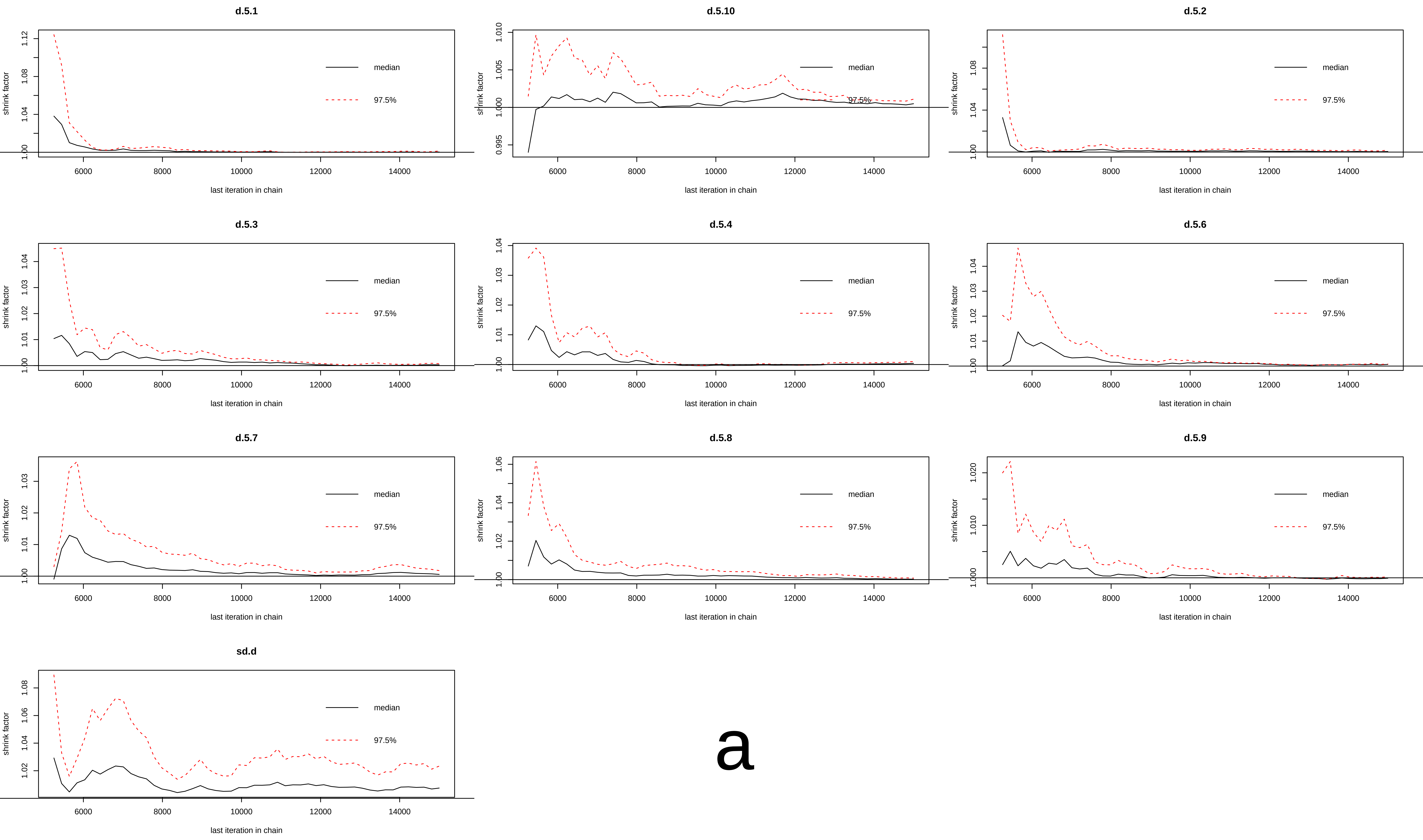
d.5.1
d.5.10
d.5.2
d.5.3
d.5.4
d.5.6
d.5.7
d.5.8
d.5.9
sd.d
shrink factor
last iteration in chain
median
97.5%

a

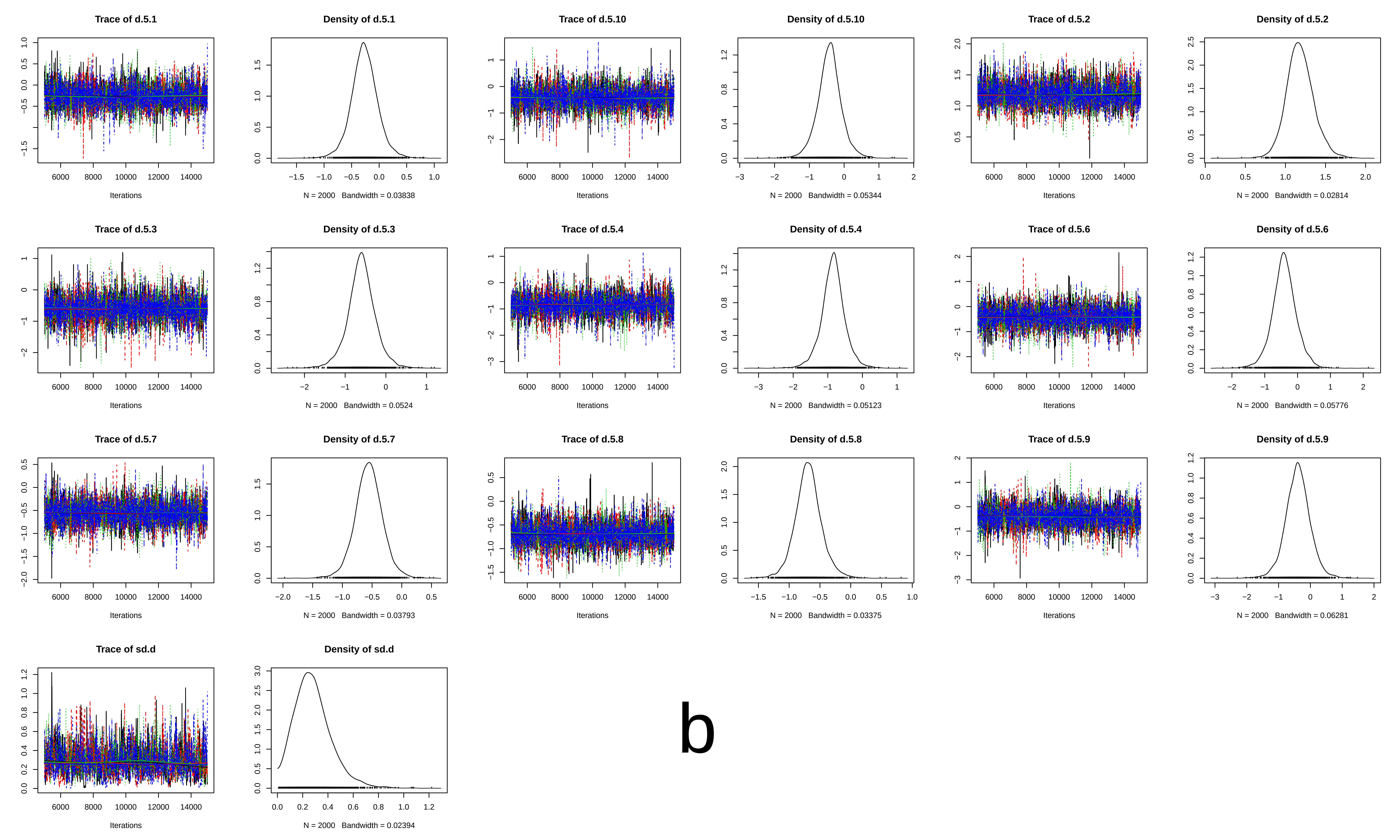
Trace of d.5.1
Iterations
Density of d.5.1
N = 2000 Bandwidth = 0.03838
Trace of d.5.10
Iterations
Density of d.5.10
N = 2000 Bandwidth = 0.05344
Trace of d.5.2
Iterations
Density of d.5.2
N = 2000 Bandwidth = 0.02814
Trace of d.5.3
Iterations
Density of d.5.3
N = 2000 Bandwidth = 0.0524
Trace of d.5.4
Iterations
Density of d.5.4
N = 2000 Bandwidth = 0.05123
Trace of d.5.6
Iterations
Density of d.5.6
N = 2000 Bandwidth = 0.05776
Trace of d.5.7
Iterations
Density of d.5.7
N = 2000 Bandwidth = 0.03793
Trace of d.5.8
Iterations
Density of d.5.8
N = 2000 Bandwidth = 0.03375
Trace of d.5.9
Iterations
Density of d.5.9
N = 2000 Bandwidth = 0.06281
Trace of sd.d
Iterations
Density of sd.d
N = 2000 Bandwidth = 0.02394
b

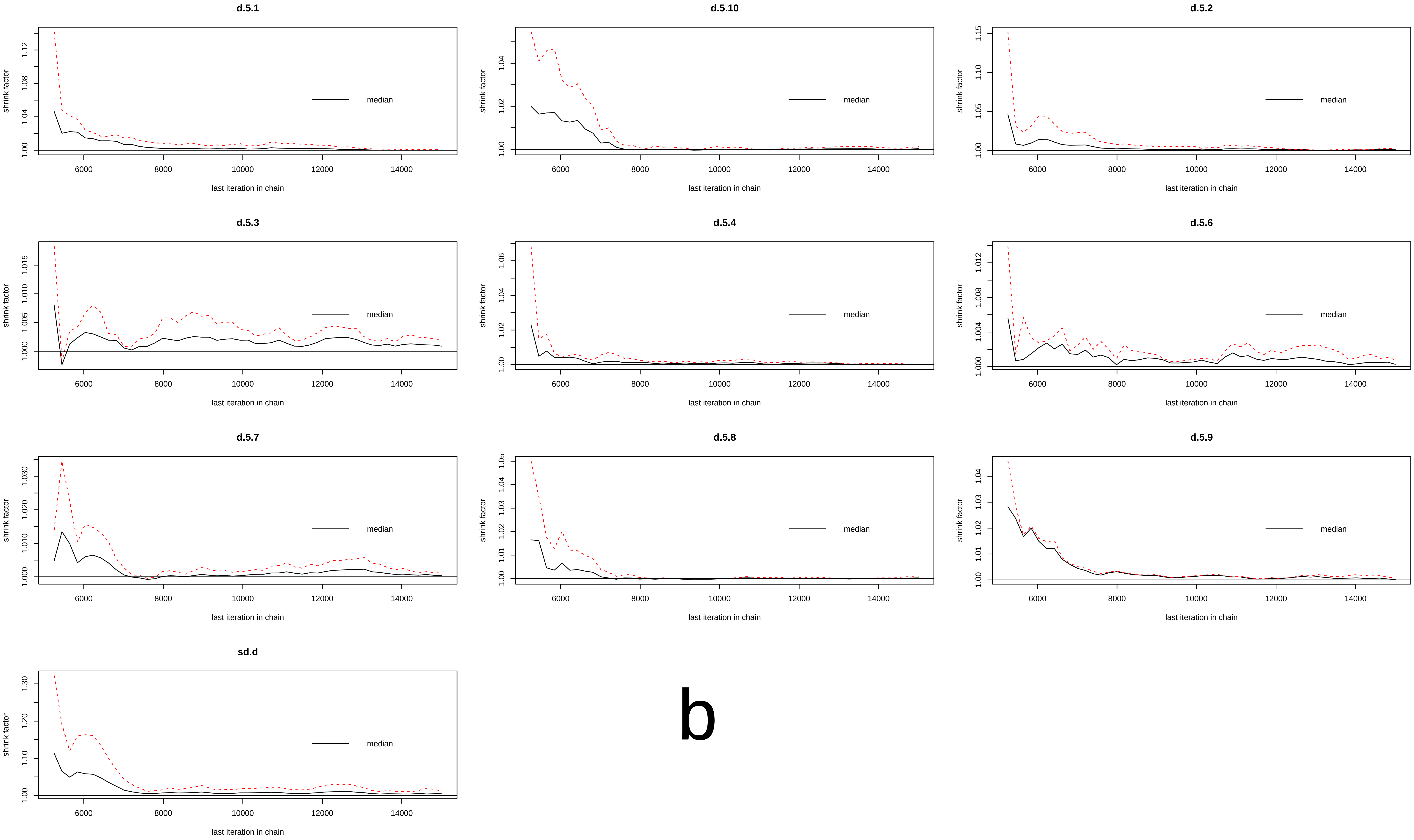
d.5.1
d.5.10
d.5.2
d.5.3
d.5.4
d.5.6
d.5.7
d.5.8
d.5.9
sd.d
shrink factor
last iteration in chain
median
b

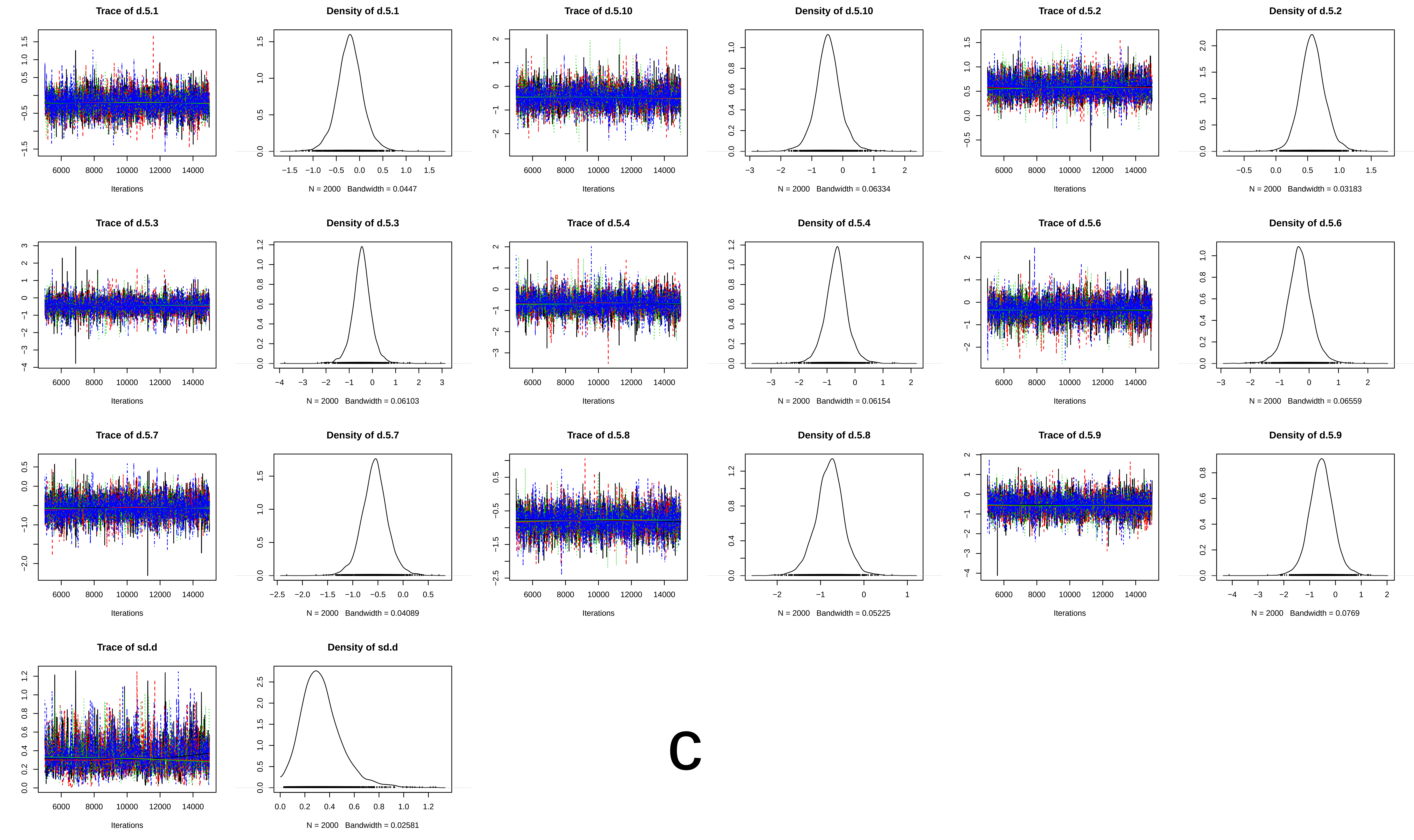
Trace of d.5.1
Iterations
Density of d.5.1
N = 2000 Bandwidth = 0.0447
Trace of d.5.10
Iterations
Density of d.5.10
N = 2000 Bandwidth = 0.06334
Trace of d.5.2
Iterations
Density of d.5.2
N = 2000 Bandwidth = 0.03183
Trace of d.5.3
Iterations
Density of d.5.3
N = 2000 Bandwidth = 0.06103
Trace of d.5.4
Iterations
Density of d.5.4
N = 2000 Bandwidth = 0.06154
Trace of d.5.6
Iterations
Density of d.5.6
N = 2000 Bandwidth = 0.06559
Trace of d.5.7
Iterations
Density of d.5.7
N = 2000 Bandwidth = 0.04089
Trace of d.5.8
Iterations
Density of d.5.8
N = 2000 Bandwidth = 0.05225
Trace of d.5.9
Iterations
Density of d.5.9
N = 2000 Bandwidth = 0.0769
Trace of sd.d
Iterations
Density of sd.d
N = 2000 Bandwidth = 0.02581

C

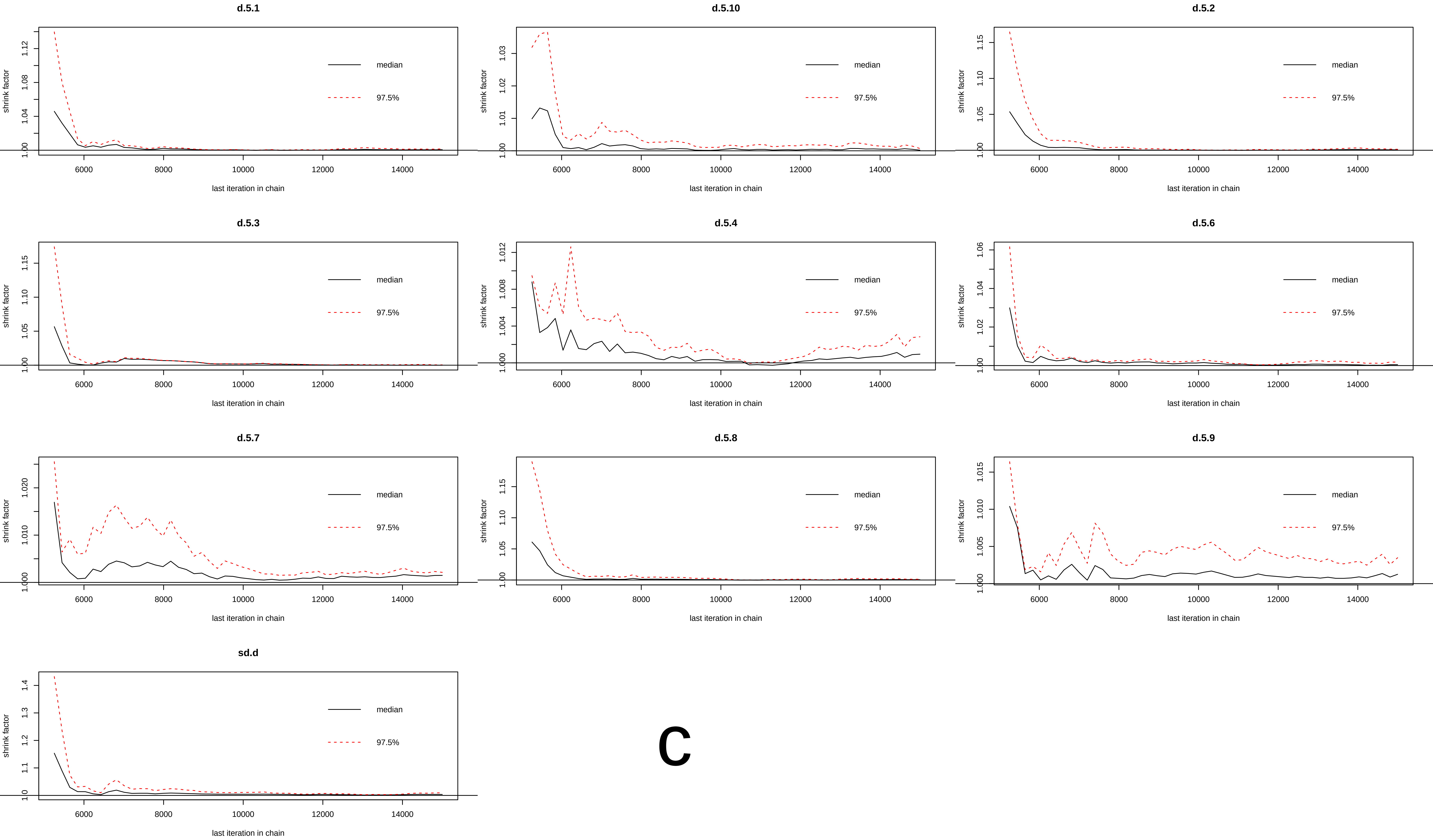
d.5.1
d.5.10
d.5.2
d.5.3
d.5.4
d.5.6
d.5.7
d.5.8
d.5.9
sd.d
shrink factor
last iteration in chain
median
97.5%
6000
8000
10000
12000
14000

C

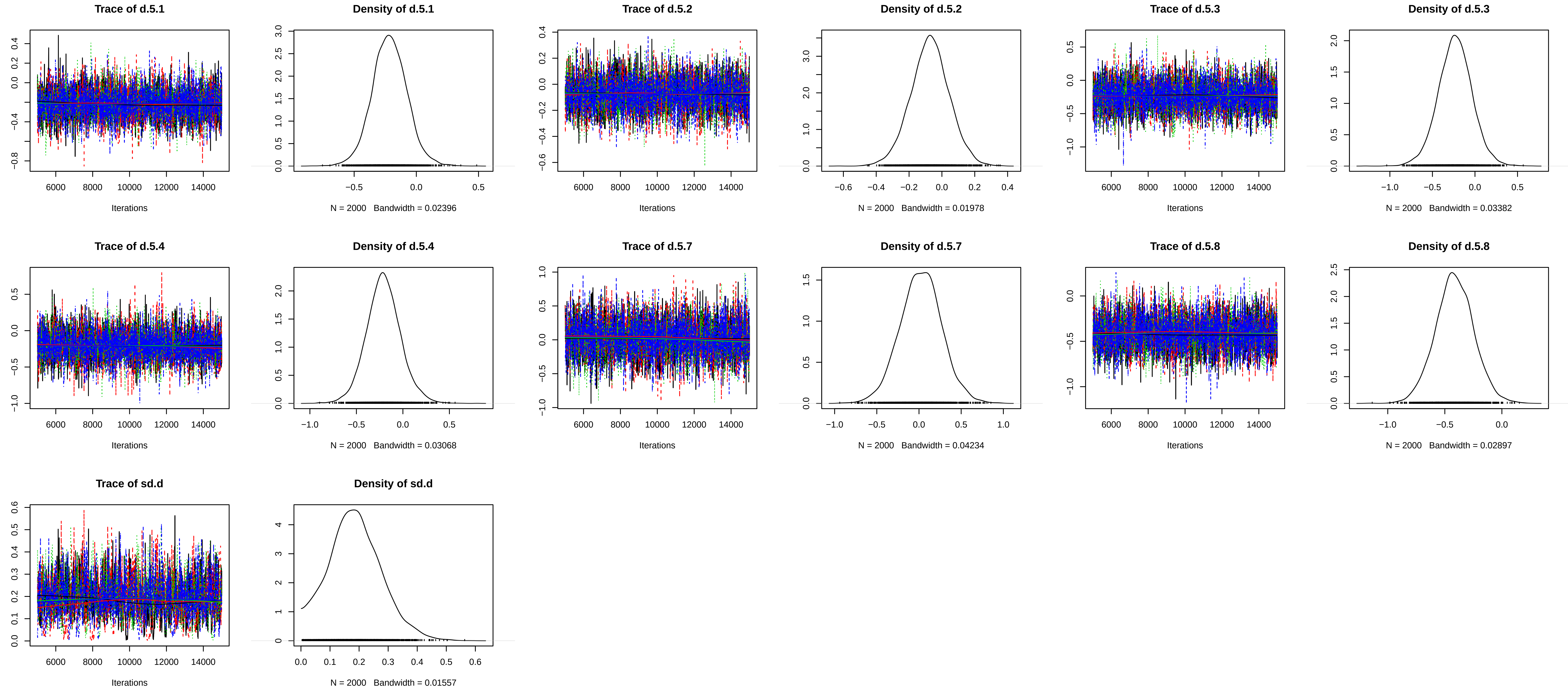
Trace of d.5.1
Iterations
Density of d.5.1
N = 2000 Bandwidth = 0.02396
Trace of d.5.2
Iterations
Density of d.5.2
N = 2000 Bandwidth = 0.01978
Trace of d.5.3
Iterations
Density of d.5.3
N = 2000 Bandwidth = 0.03382
Trace of d.5.4
Iterations
Density of d.5.4
N = 2000 Bandwidth = 0.03068
Trace of d.5.7
Iterations
Density of d.5.7
N = 2000 Bandwidth = 0.04234
Trace of d.5.8
Iterations
Density of d.5.8
N = 2000 Bandwidth = 0.02897
Trace of sd.d
Iterations
Density of sd.d
N = 2000 Bandwidth = 0.01557

d

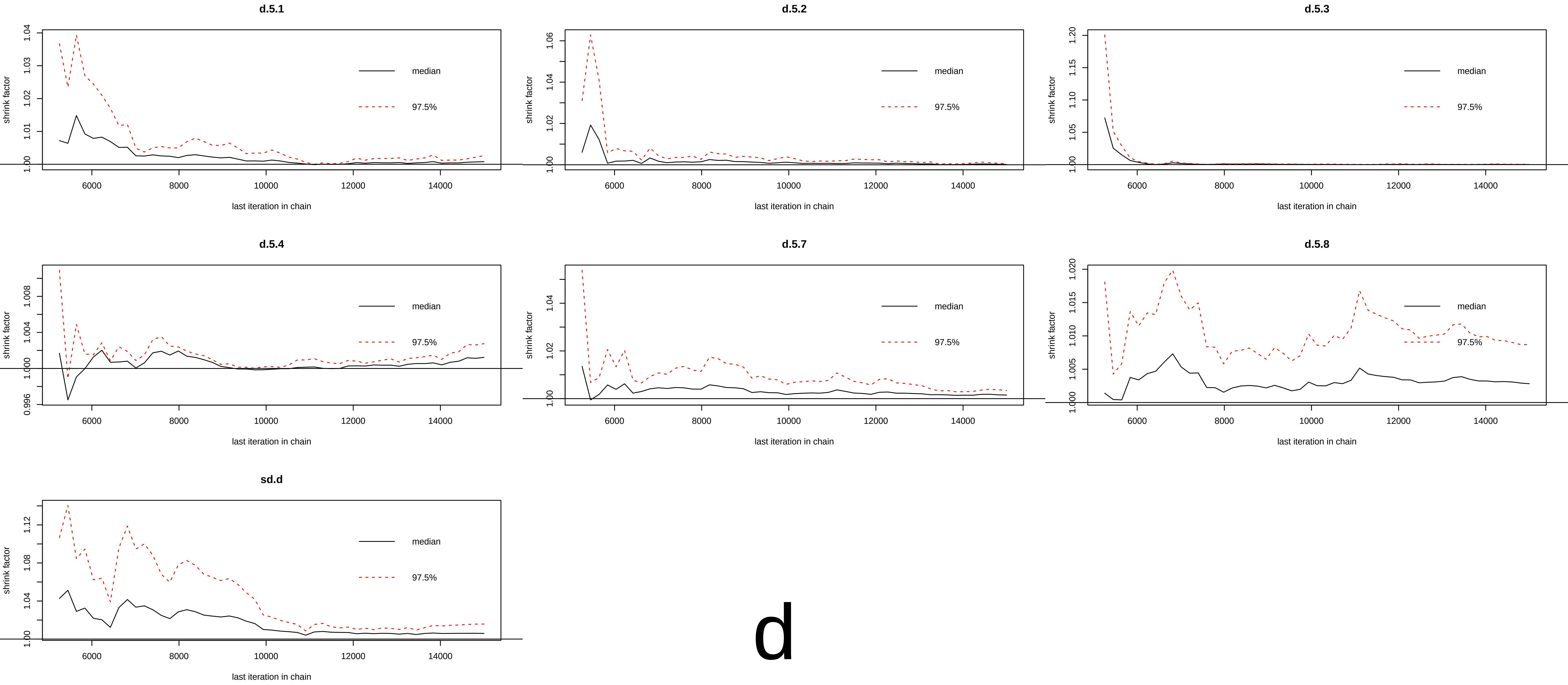
d.5.1
d.5.2
d.5.3
d.5.4
d.5.7
d.5.8
sd.d
shrink factor
last iteration in chain
median
97.5%
6000
8000
10000
12000
14000

d

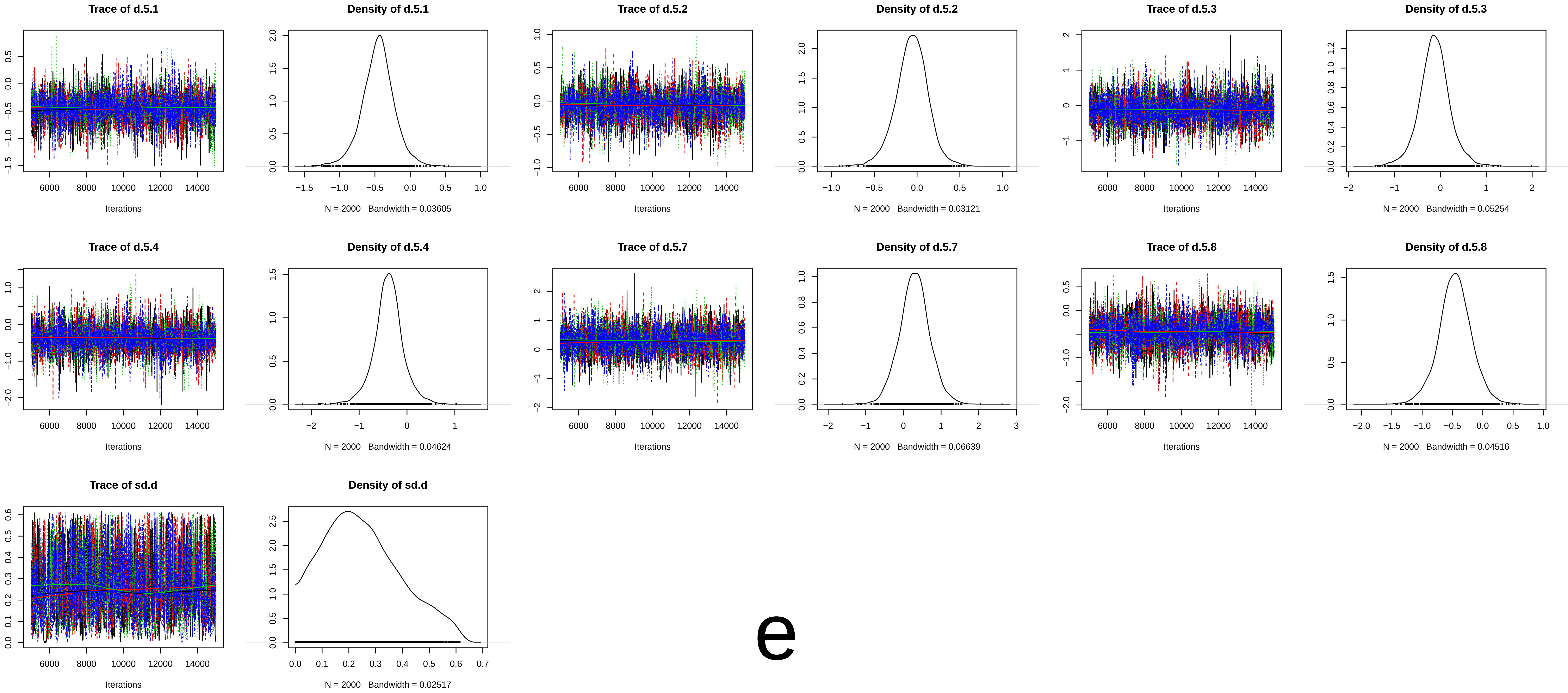

Trace of d.5.1
Density of d.5.1
Trace of d.5.2
Density of d.5.2
Trace of d.5.3
Density of d.5.3
Iterations
N = 2000 Bandwidth = 0.03605
Iterations
N = 2000 Bandwidth = 0.03121
Iterations
N = 2000 Bandwidth = 0.05254
Trace of d.5.4
Density of d.5.4
Trace of d.5.7
Density of d.5.7
Trace of d.5.8
Density of d.5.8
Iterations
N = 2000 Bandwidth = 0.04624
Iterations
N = 2000 Bandwidth = 0.06639
Iterations
N = 2000 Bandwidth = 0.04516
Trace of sd.d
Density of sd.d
Iterations
N = 2000 Bandwidth = 0.02517

e

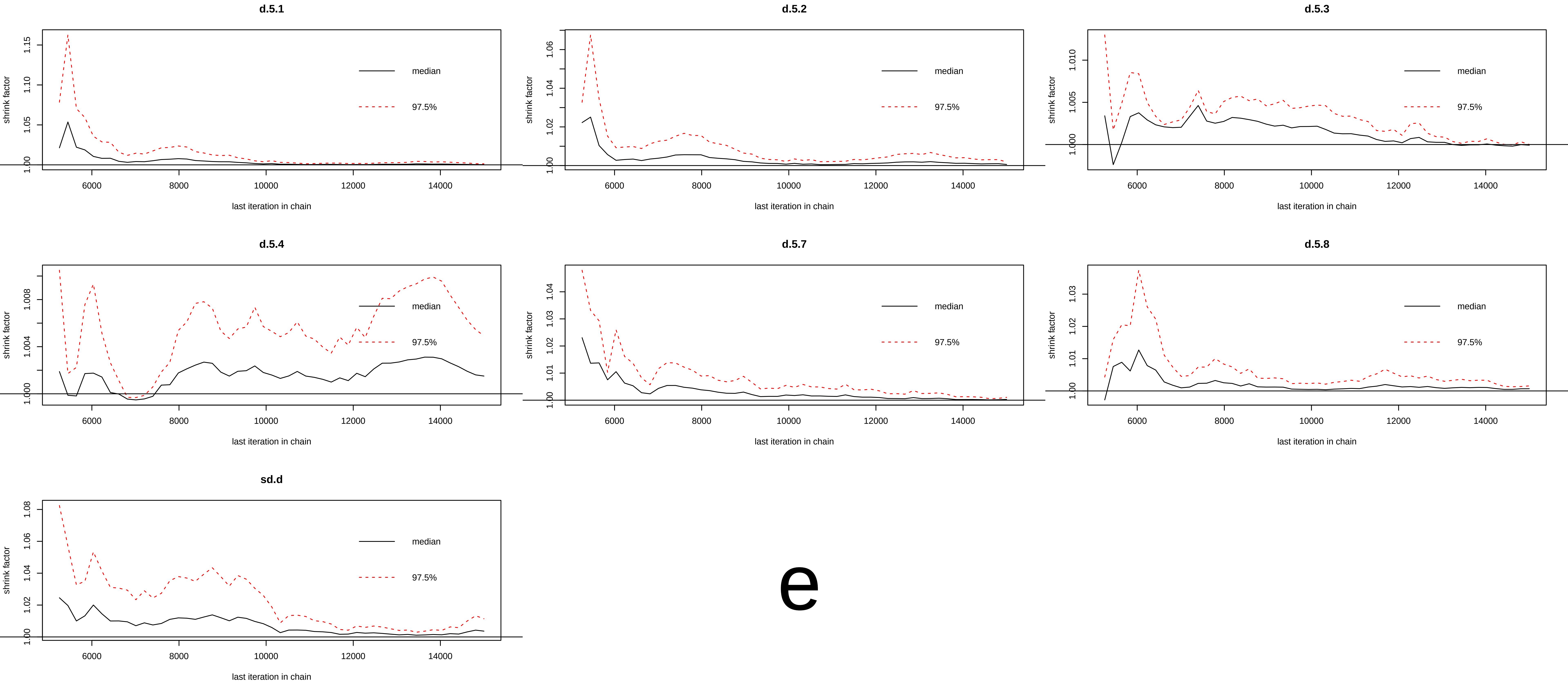
d.5.1
d.5.2
d.5.3
d.5.4
d.5.7
d.5.8
sd.d
shrink factor
last iteration in chain
median
97.5%
6000
8000
10000
12000
14000

e

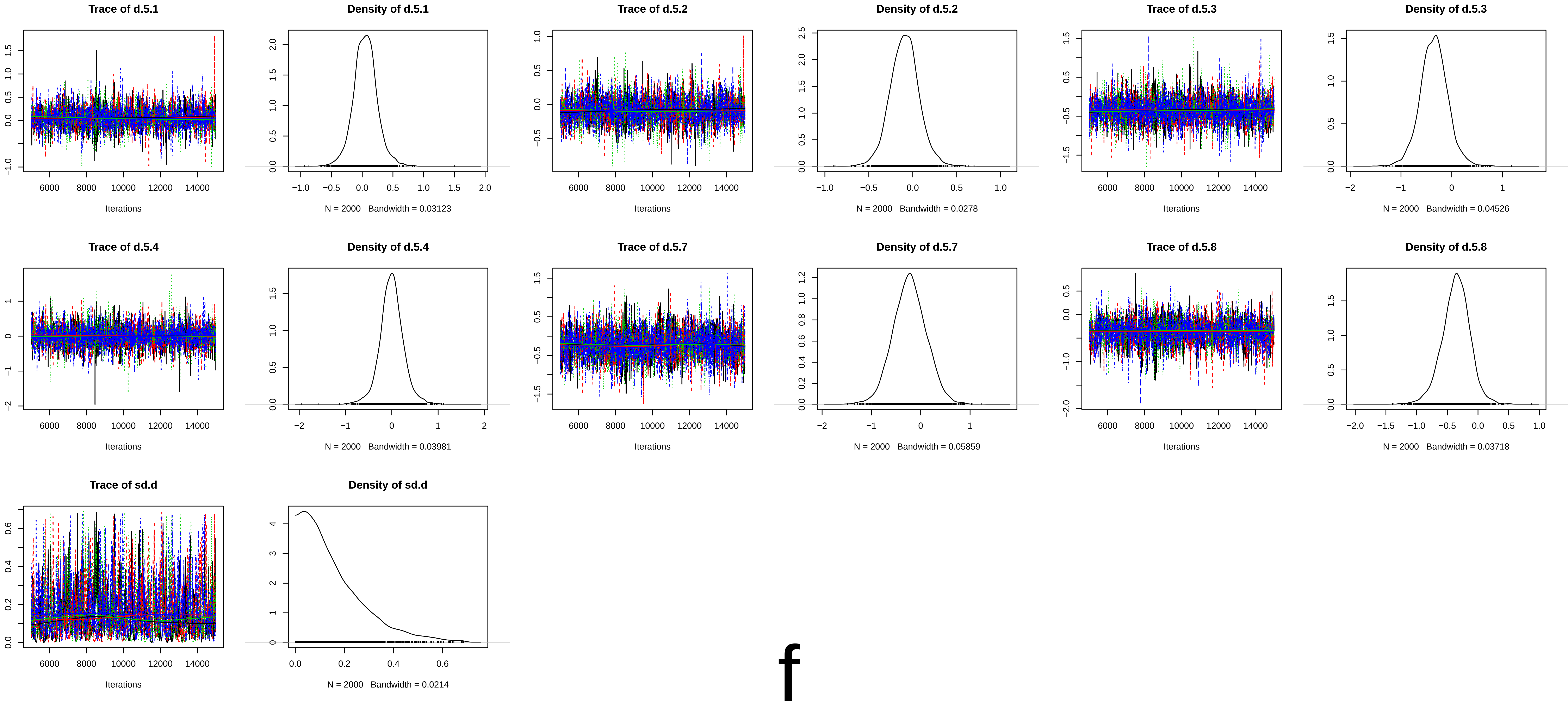
Trace of d.5.1
Iterations
Density of d.5.1
N = 2000 Bandwidth = 0.03123
Trace of d.5.2
Iterations
Density of d.5.2
N = 2000 Bandwidth = 0.0278
Trace of d.5.3
Iterations
Density of d.5.3
N = 2000 Bandwidth = 0.04526
Trace of d.5.4
Iterations
Density of d.5.4
N = 2000 Bandwidth = 0.03981
Trace of d.5.7
Iterations
Density of d.5.7
N = 2000 Bandwidth = 0.05859
Trace of d.5.8
Iterations
Density of d.5.8
N = 2000 Bandwidth = 0.03718
Trace of sd.d
Iterations
Density of sd.d
N = 2000 Bandwidth = 0.0214

f

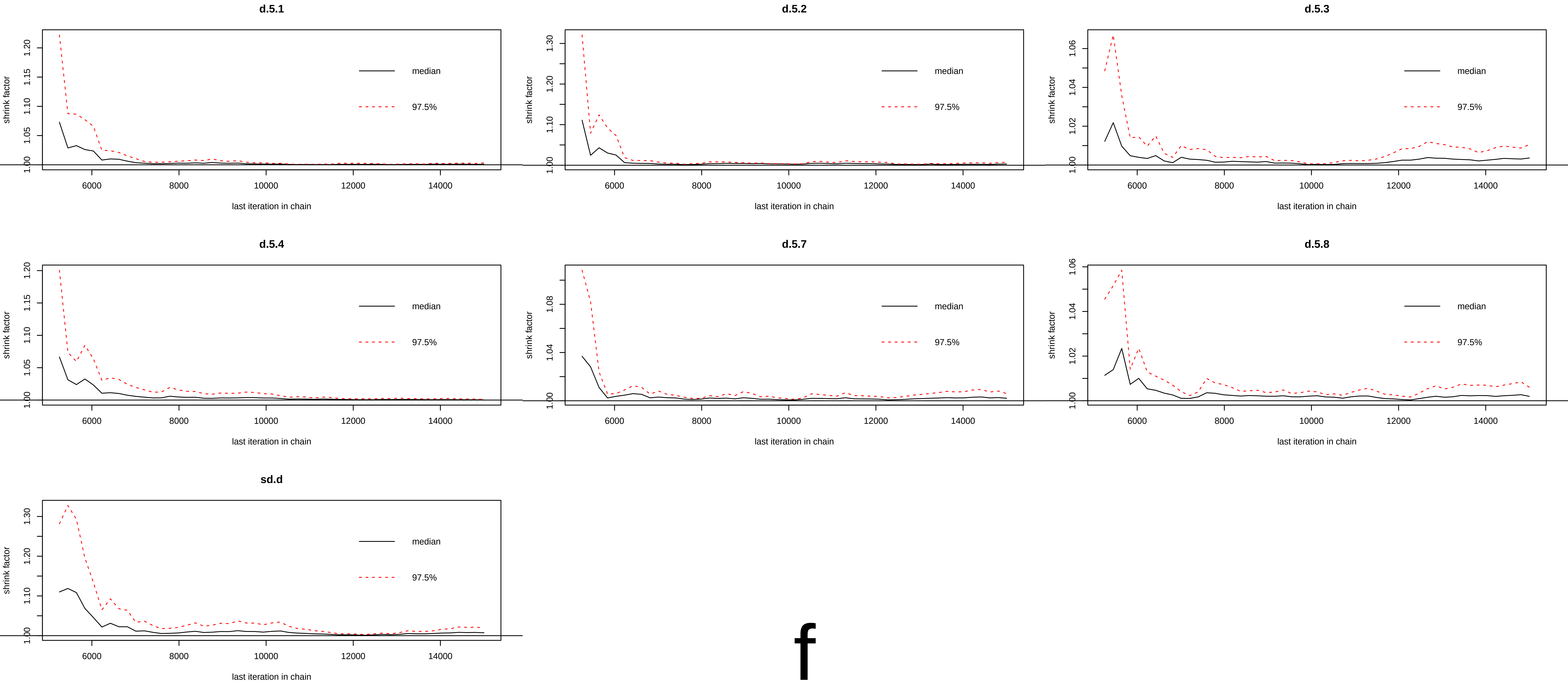
d.5.1
d.5.2
d.5.3
d.5.4
d.5.7
d.5.8
sd.d
shrink factor
last iteration in chain
median
97.5%
6000
8000
10000
12000
14000

f
