# Supplementary material for: Front-Line Therapy in EGFR Exon 19 Deletion and 21 Leu858Arg Mutations in Advanced Non-Small Cell Lung Cancer: A Network Meta-Analysis
Source: Evid Based Complement Alternat Med. 2021 Dec 13;2021:9311875. doi: 10.1155/2021/9311875 (PMC8687779; doi:10.1155/2021/9311875)
Supplement: Supplementary Materials — There are 4 supplementary tables and 2 supplementary figures for this paper. [file 9311875.f1.zip › 9311875.f1/FigureS2 (1).pdf]

Figure S2 Bayesian ranking profiles of comparable treatments on efficacy for patients with non-small cell lung cancer.

Profiles indicate the probability of each comparable treatment being ranked from first to last on progression free survival (PFS) and overall survival (OS). SoC: standard of care, represents first generation EGFR-TKIs in this network meta-analysis (including gefitinib, erlotinib and icotinib).

a. Afatinib; b. Chemotherapy; c. Dacomitinib; d. Osimertinib; e. SoC; f. SoC+Apatinib; g. SoC+Bevacizumab; h. SoC+Chemotherapy; i. SoC+Mono−chemotherapy; j.SoC+Ramucirumab

a

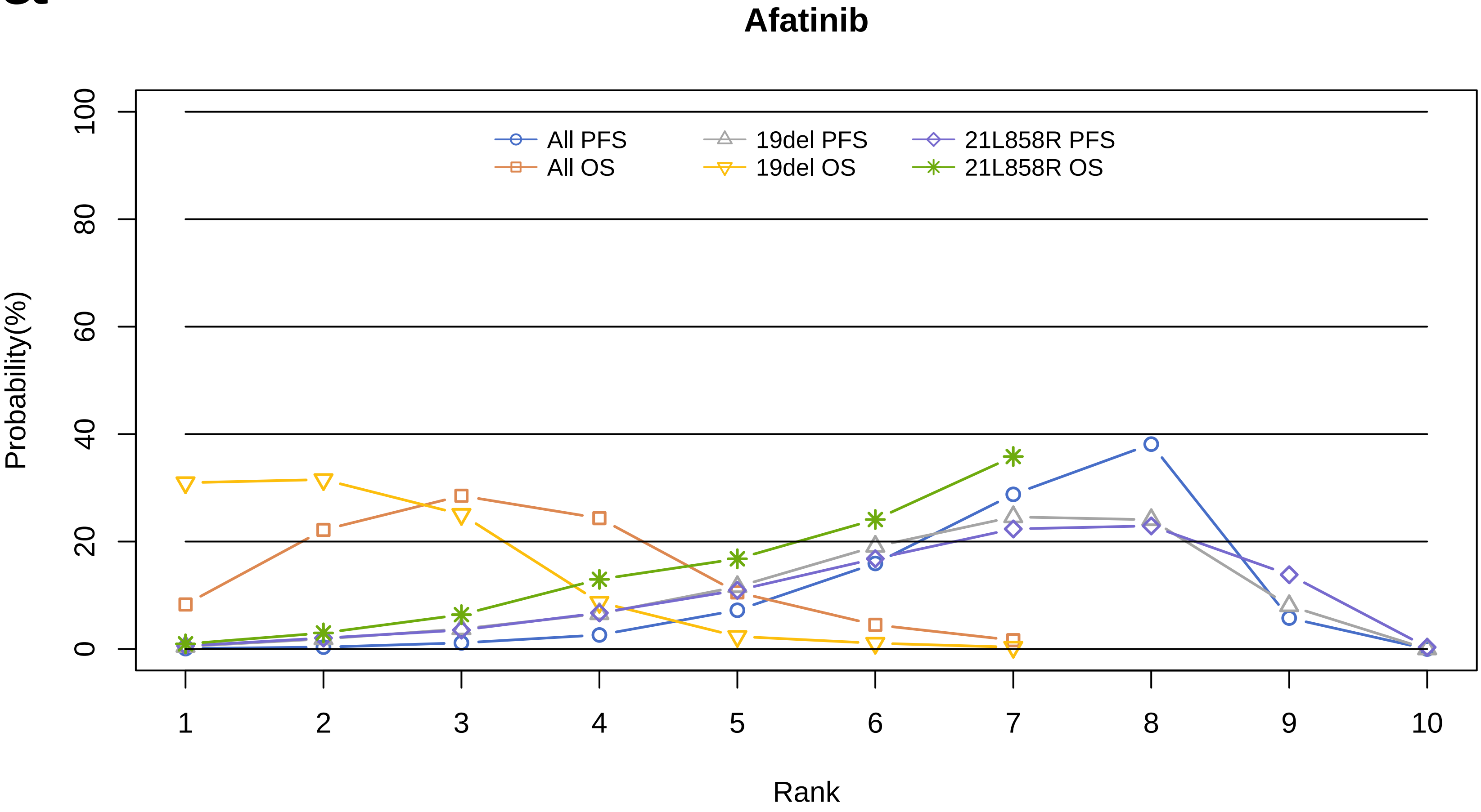

Afatinib
All PFS
All OS
19del PFS
19del OS
21L858R PFS
21L858R OS
Probability(%)
0
20
40
60
80
100
Rank
1
2
3
4
5
6
7
8
9
10

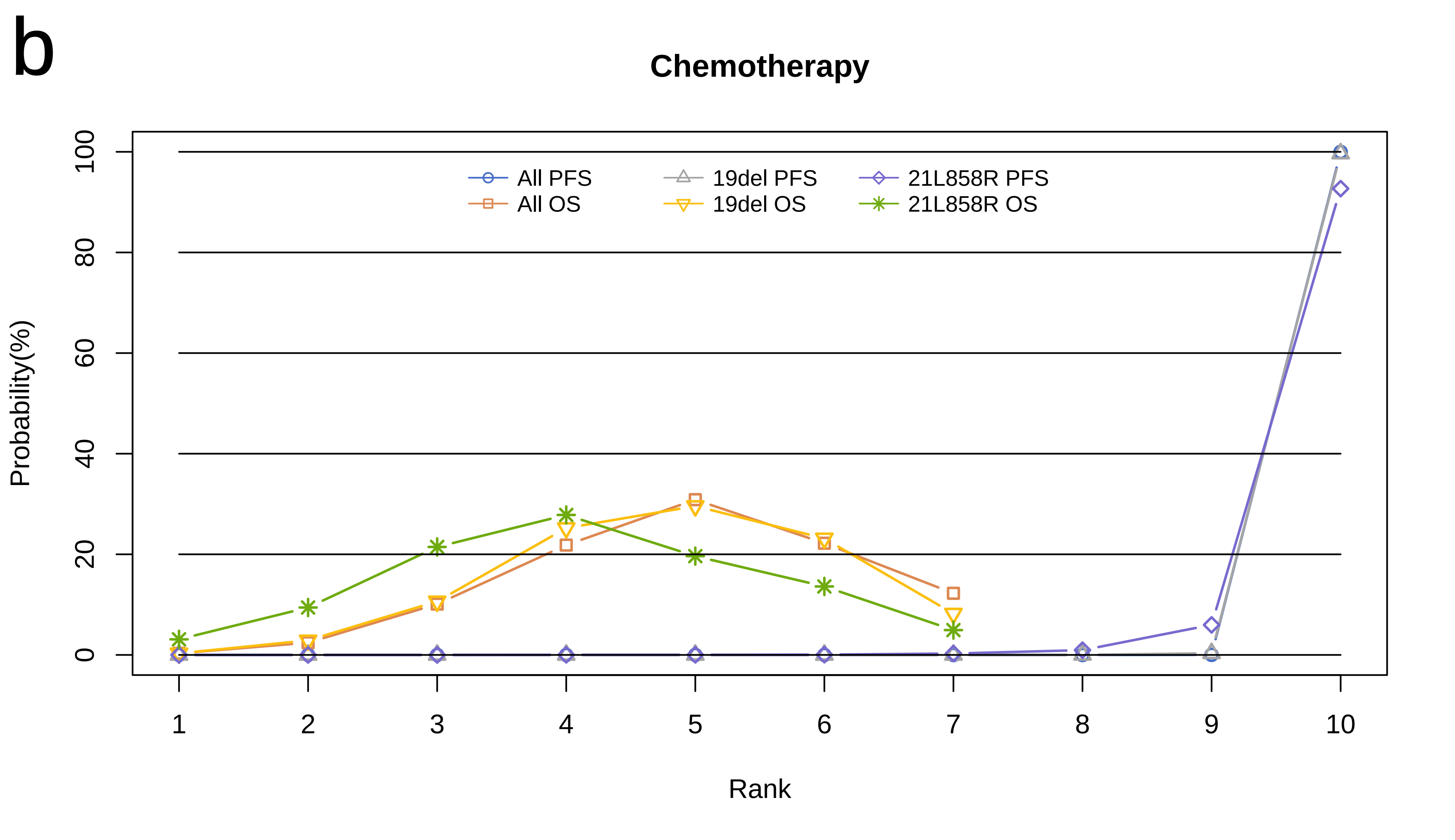
b
Chemotherapy
All PFS
All OS
19del PFS
19del OS
21L858R PFS
21L858R OS
Probability(%)
0
20
40
60
80
100
Rank
1
2
3
4
5
6
7
8
9
10

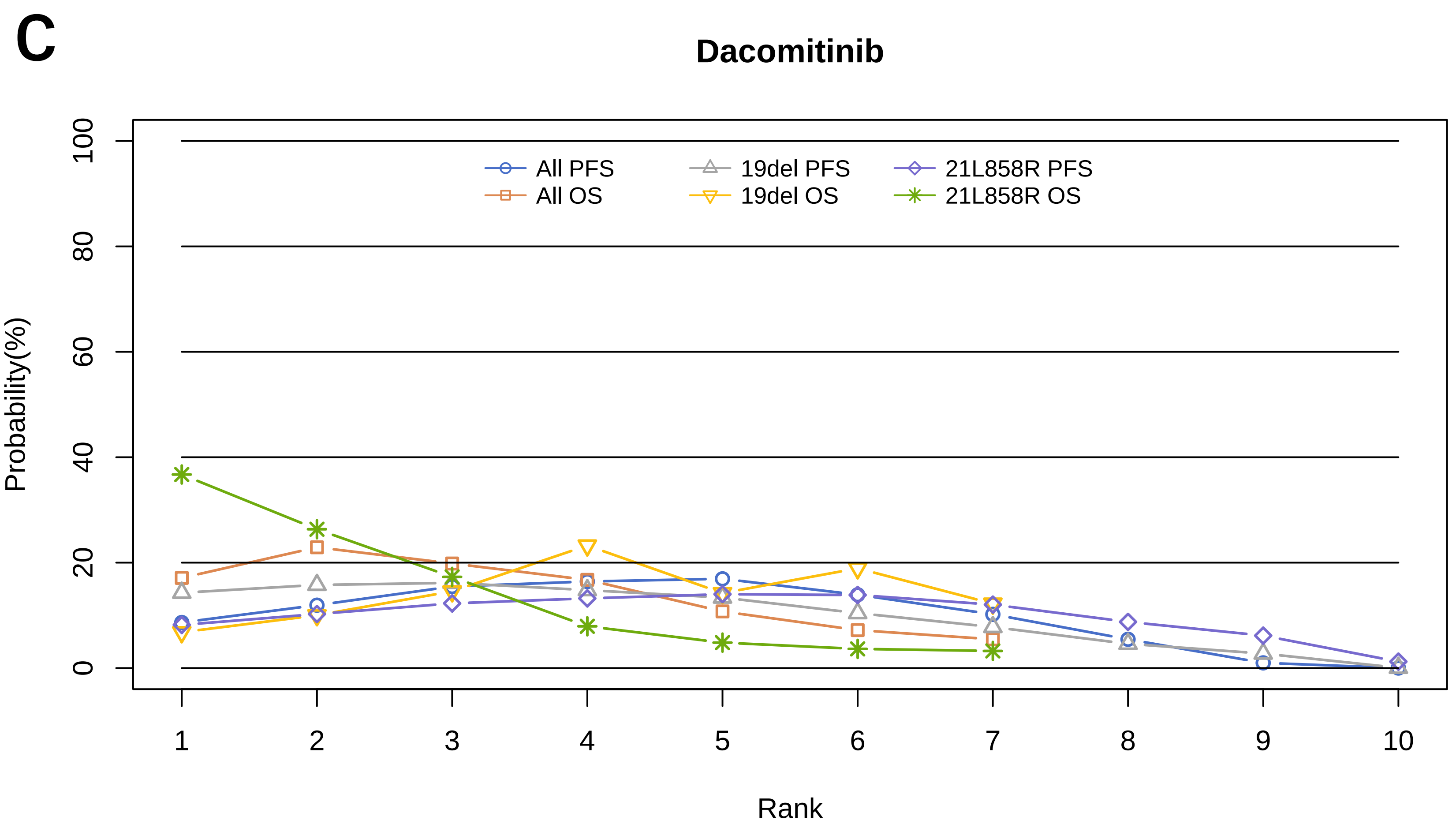

C
Dacomitinib
All PFS
All OS
19del PFS
19del OS
21L858R PFS
21L858R OS
Probability(%)
0
20
40
60
80
100
1
2
3
4
5
6
7
8
9
10
Rank

d

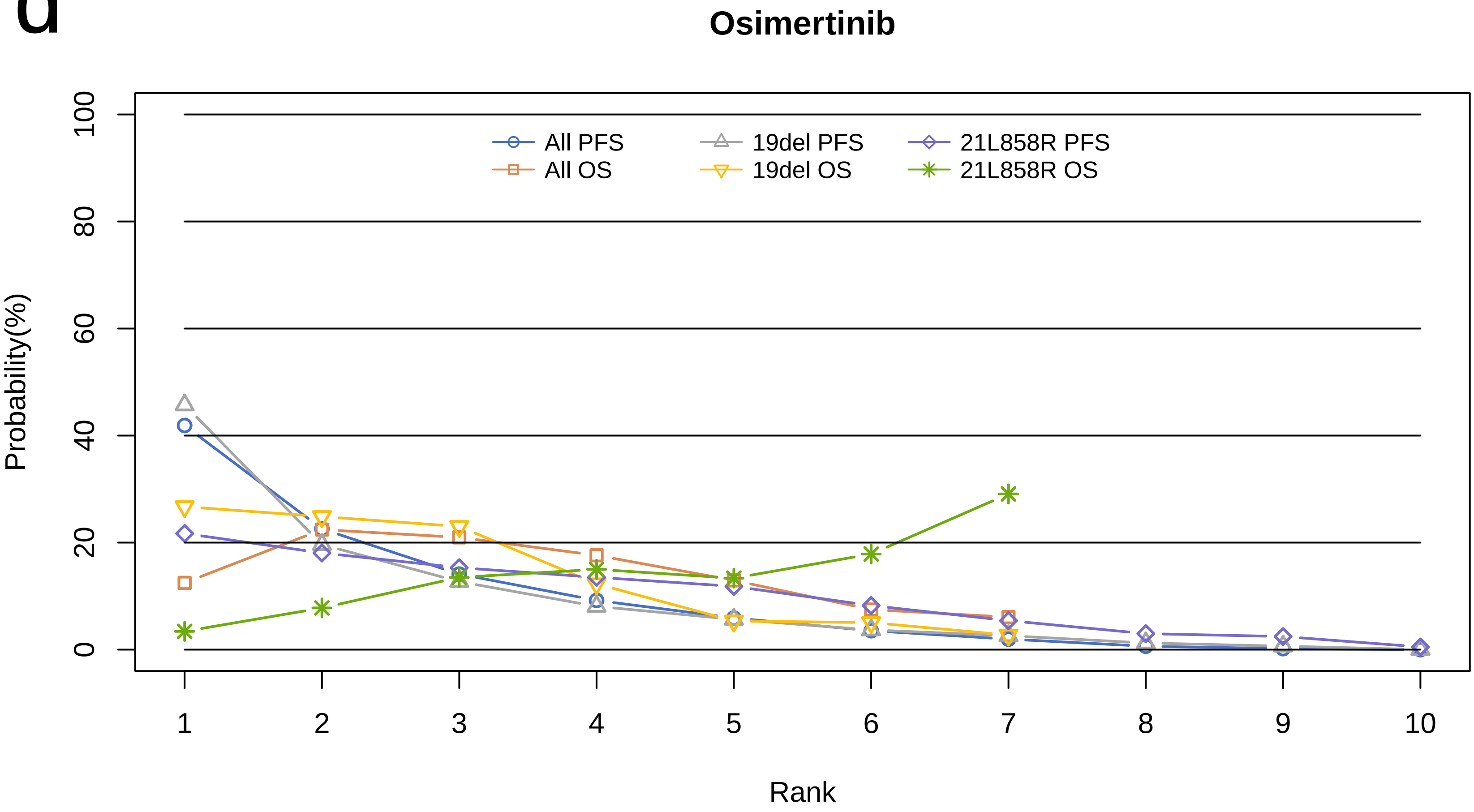
Osimertinib
All PFS
All OS
19del PFS
19del OS
21L858R PFS
21L858R OS
Probability(%)
100
80
60
40
20
0
1
2
3
4
5
6
7
8
9
10
Rank

e

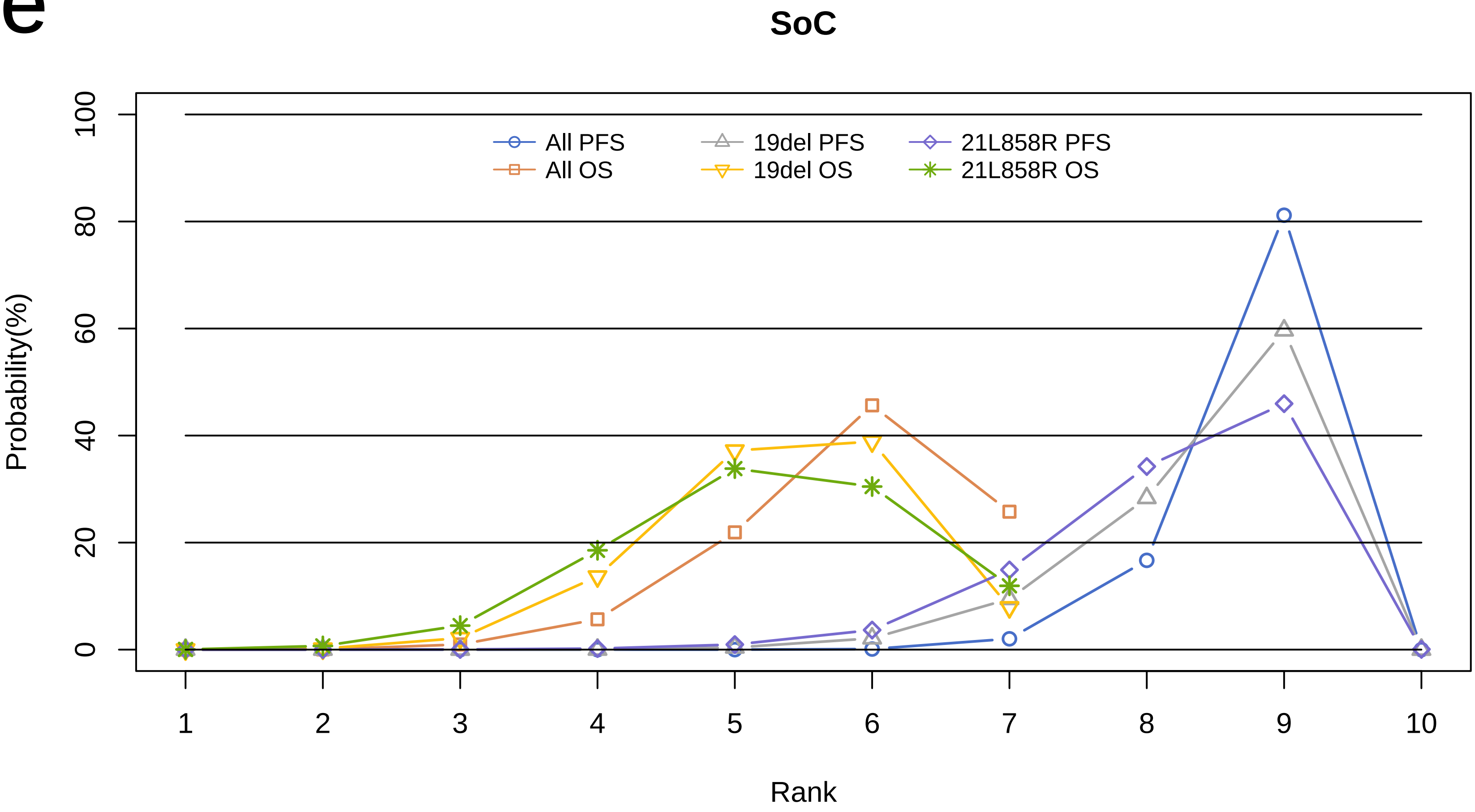
SoC
All PFS
All OS
19del PFS
19del OS
21L858R PFS
21L858R OS
Probability(%)
0
20
40
60
80
100
Rank
1
2
3
4
5
6
7
8
9
10

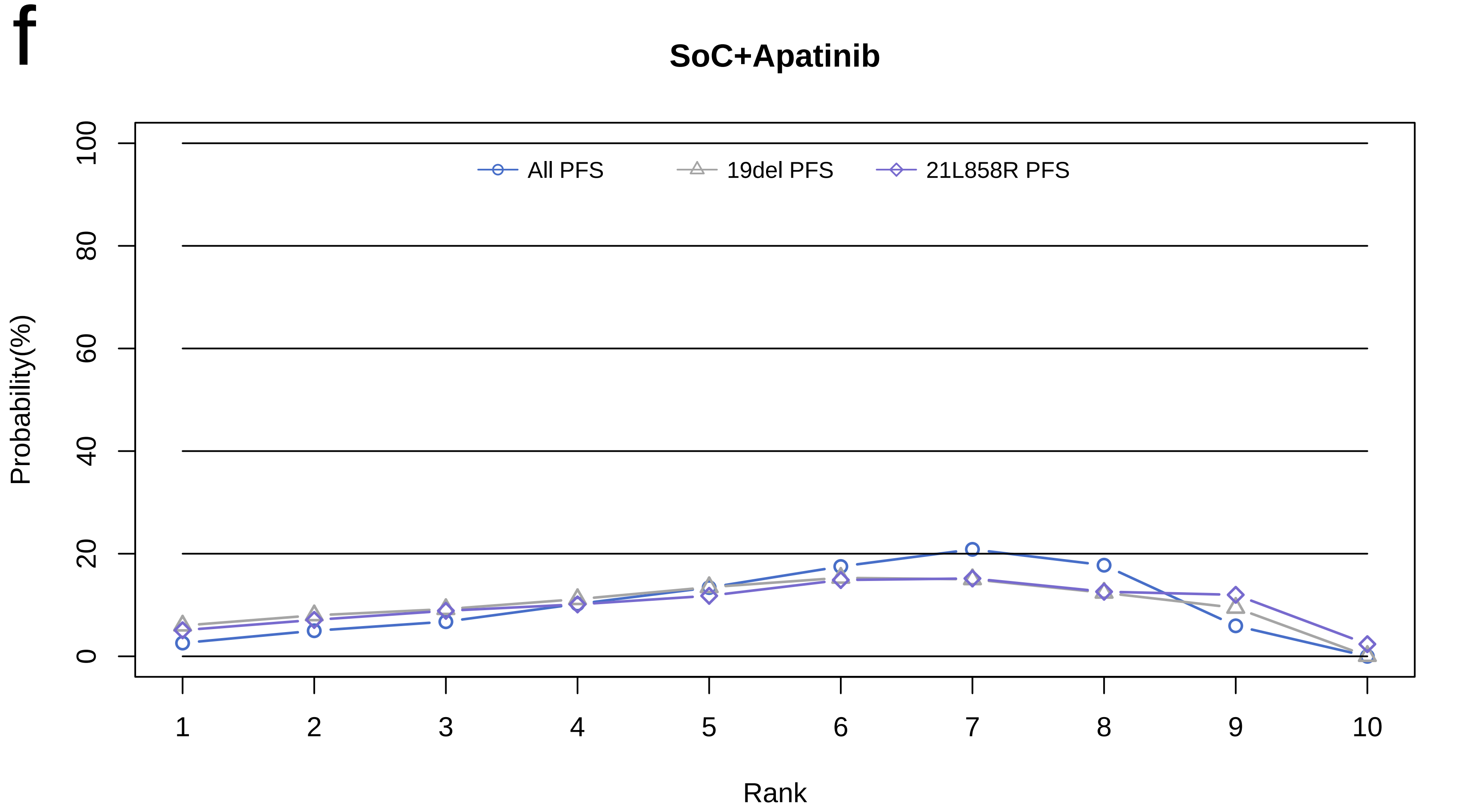
f
SoC+Apatinib
All PFS
19del PFS
21L858R PFS
Probability(%)
0
20
40
60
80
100
1
2
3
4
5
6
7
8
9
10
Rank

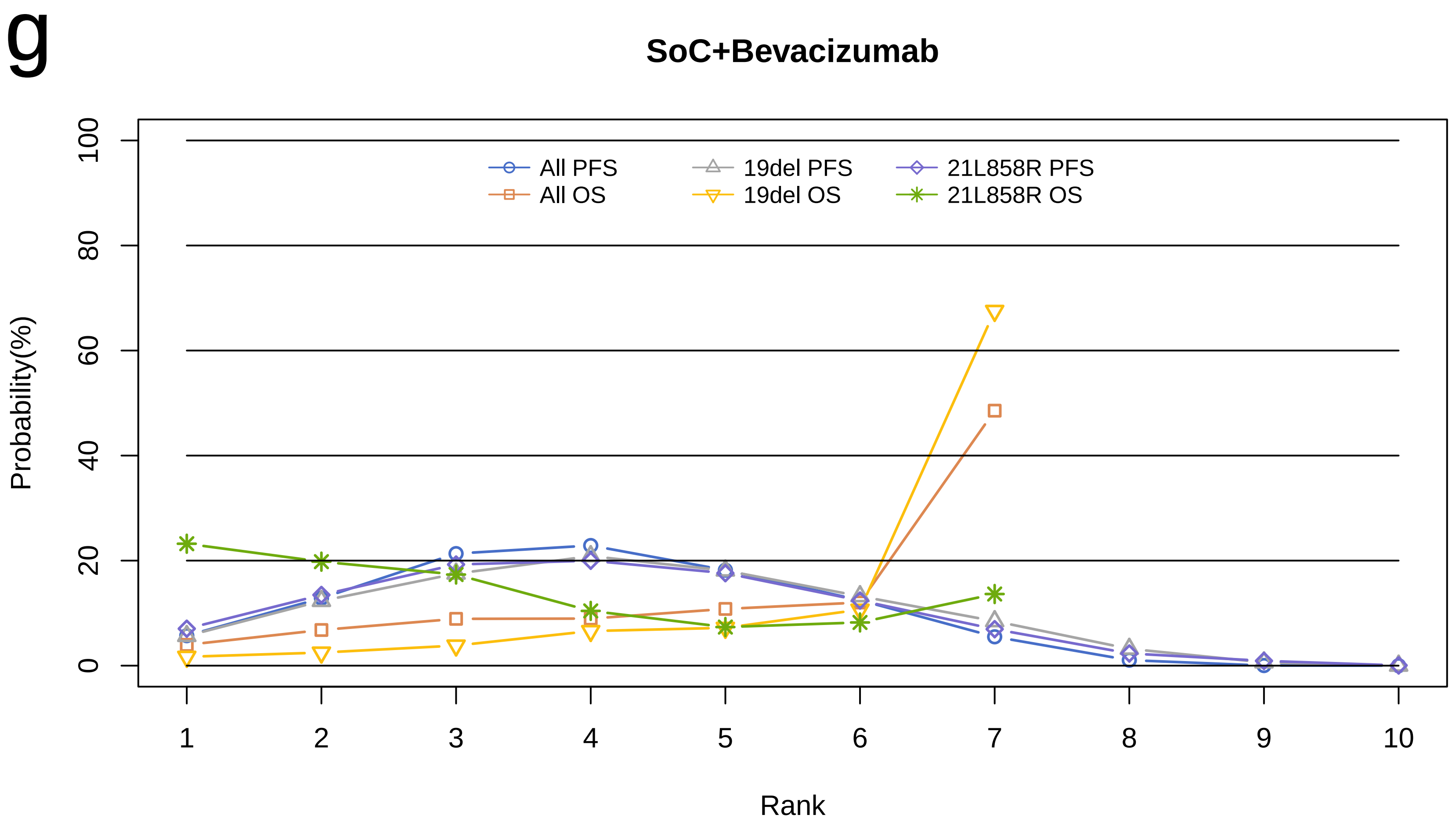
g
SoC+Bevacizumab
All PFS
All OS
19del PFS
19del OS
21L858R PFS
21L858R OS
Probability(%)
0
20
40
60
80
100
1
2
3
4
5
6
7
8
9
10
Rank

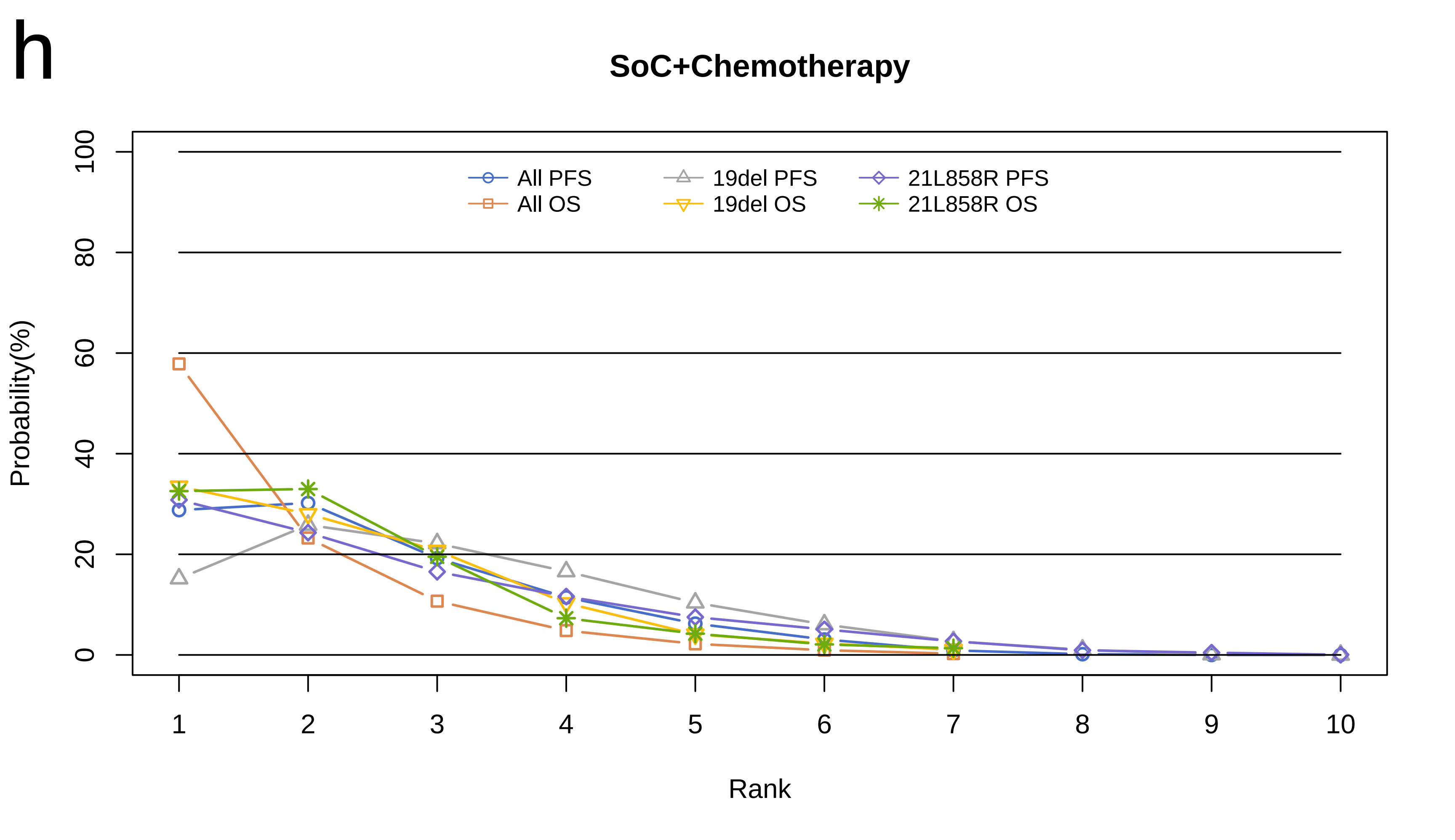
h
SoC+Chemotherapy
All PFS
All OS
19del PFS
19del OS
21L858R PFS
21L858R OS
Probability(%)
0
20
40
60
80
100
Rank
1
2
3
4
5
6
7
8
9
10

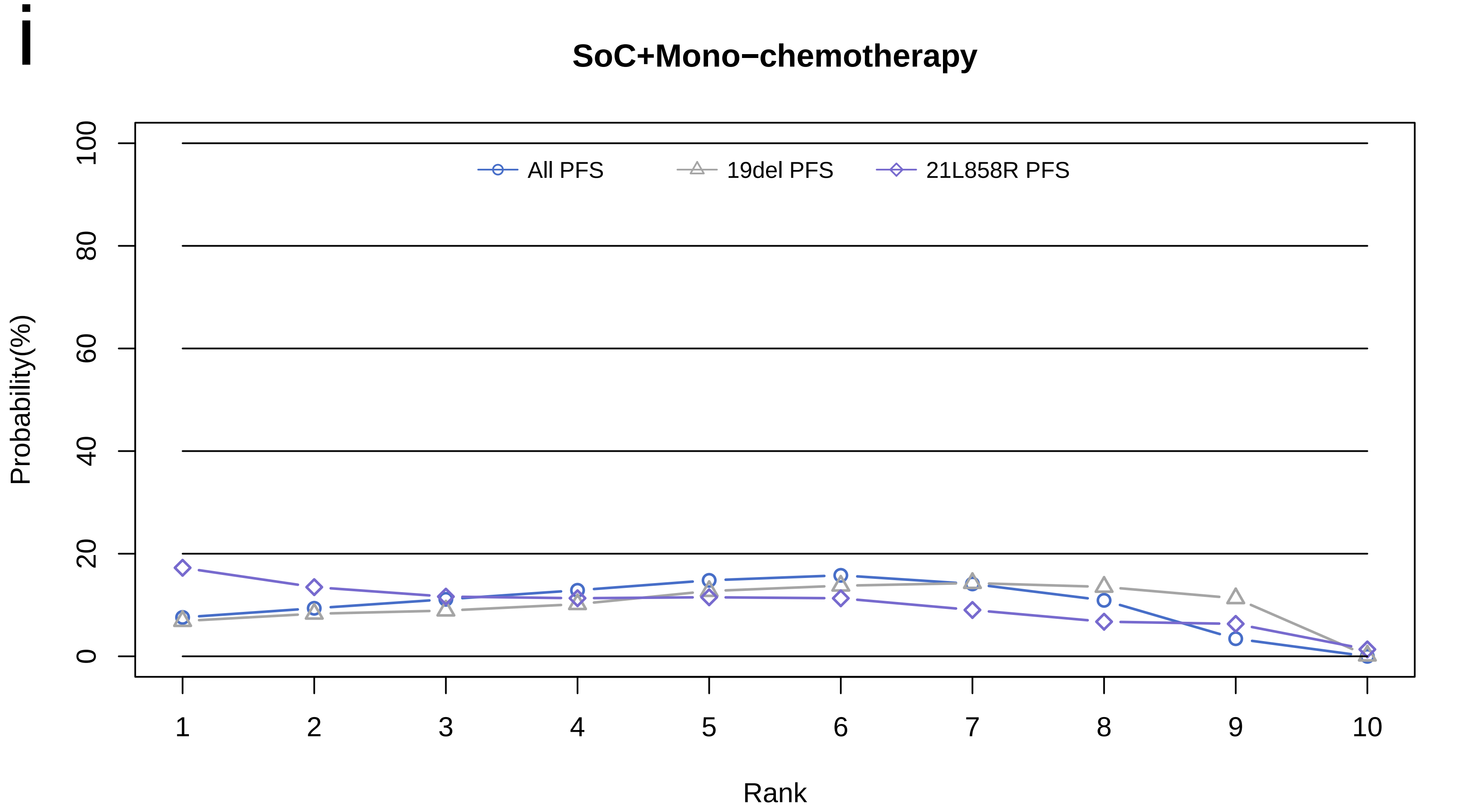
i
SoC+Mono−chemotherapy
All PFS
19del PFS
21L858R PFS
Probability(%)
0
20
40
60
80
100
1
2
3
4
5
6
7
8
9
10
Rank

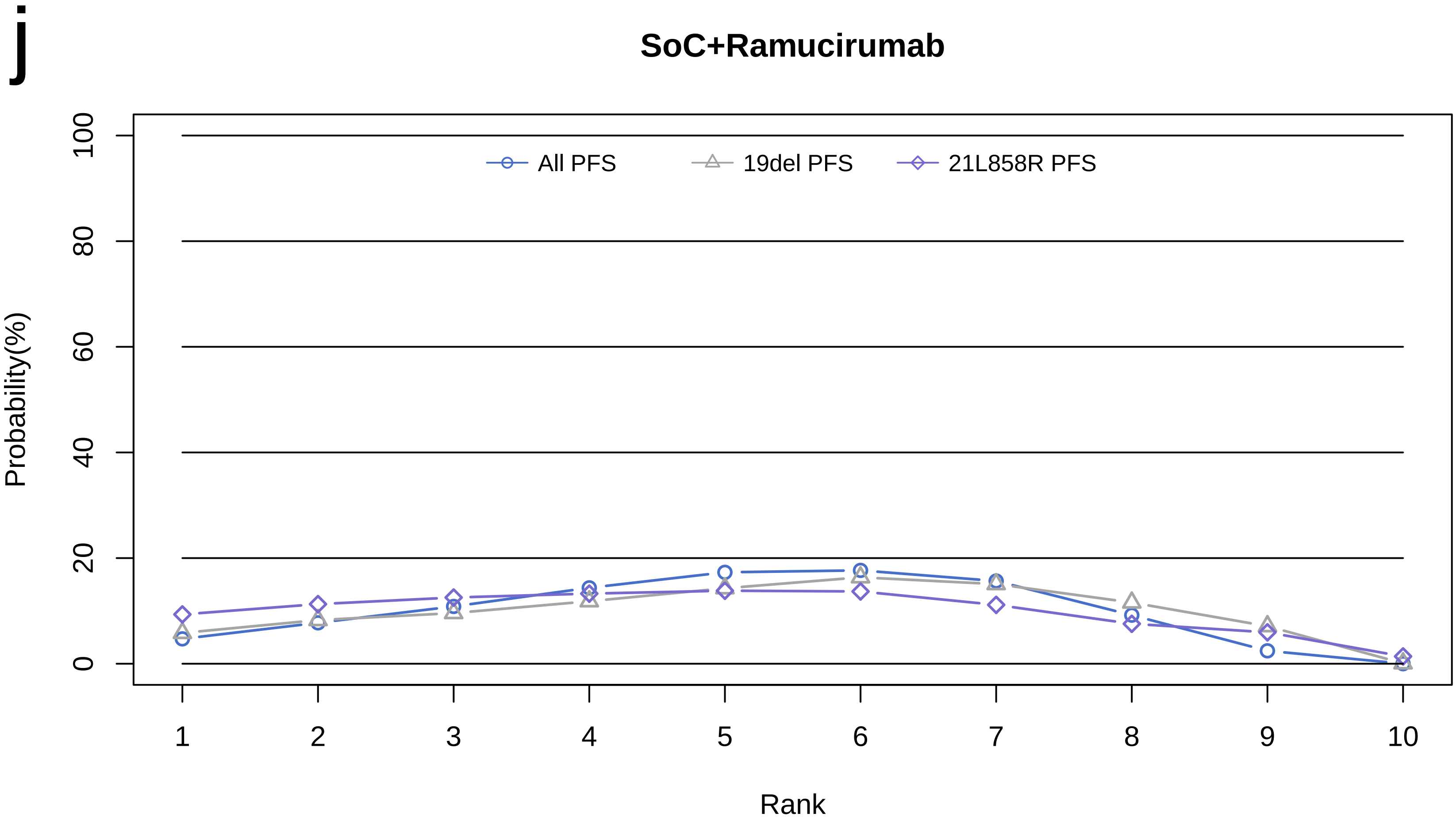
j
SoC+Ramucirumab
All PFS
19del PFS
21L858R PFS
Probability(%)
0
20
40
60
80
100
1
2
3
4
5
6
7
8
9
10
Rank
