# Supplementary material for: Front-Line Therapy in EGFR Exon 19 Deletion and 21 Leu858Arg Mutations in Advanced Non-Small Cell Lung Cancer: A Network Meta-Analysis
Source: Evid Based Complement Alternat Med. 2021 Dec 13;2021:9311875. doi: 10.1155/2021/9311875 (PMC8687779; doi:10.1155/2021/9311875)
Supplement: Supplementary Materials — There are 4 supplementary tables and 2 supplementary figures for this paper. [file 9311875.f1.zip › 9311875.f1/TableS1.pdf]

Table S1 Checklist of the PRISMA extension for network meta-analysis

International Platform of Registered Systematic Review and Meta-analysis Protocols

INPLASY PROTOCOL

To cite: Xie et al. Front-line Therapy in EGFR exon 19 deletion and Leu858Arg mutations in Advanced None-Small Cell Lung Cancer: A Network Meta-analysis. Inplasy protocol 2020100059. doi: 10.37766/inplasy2020.10.0059

Received: 17 October 2020

Published: 17 October 2020

Corresponding author:
Junling Li

lijunling@cicams.ac.cn

Author Affiliation:
Cancer Hospital, Chinese Academy of Medical Sciences and Peking Union Medical College, Beijing 100021, China

Support: **None.**

Review Stage at time of this submission: **The review has not yet started.**

Conflicts of interest:
None.

Front-line Therapy in EGFR exon 19 deletion and Leu858Arg mutations in Advanced None-Small Cell Lung Cancer: A Network Meta-analysis

Xie, TJ[1]; Zou, ZH[2]; Liu, CC[3]; Zhu, YX[4]; Xu, ZY[5]; Wang, L[6]; Xing, PY[7]; Li, JL [8].

Review question / Objective: **Patient: Patients with non-small cell lung cancer(NSCLC) harboring epidermal growth factor receptor(EGFR) mutation; Intervention: Tyrosine kinase inhibitors(TKIs) with or without anti-VEGF; Comparison: TKIs or chemotherapy; Outcomes: OS, PFS; Study design: Randomized controlled trial.**
Condition being studied: **Lung cancer is the leading cause of cancer-related death worldwide. About 85% reported lung cancer cases are non-small-cell lung cancer(NSCLC). In the past few decades, epidermal growth factor receptor tyrosine kinase inhibitors(EGFR-TKIs) has improved the clinical prognosis of NSCLC patients, which is definitely the typical example of precision treatment. There are lots of EGFR-TKIs currently in common use, and studies of these drugs have included populations with many different baseline characteristics, including EGFR mutation type. Therefore, we performed this network meta-analysis of randomised controlled trials with latest information to analyze the optimal treatment options for EGFR exon 19 deletion and Leu858Arg mutations patients respectively.**

INPLASY registration number: This protocol was registered with the International Platform of Registered Systematic Review and Meta-Analysis Protocols (INPLASY) on 17 October 2020 and was last updated on 17 October 2020 (registration number INPLASY2020100059).

INTRODUCTION

Review question / Objective: **Patient: Patients with non-small cell lung cancer(NSCLC) harboring epidermal growth factor receptor(EGFR) mutation; Intervention: Tyrosine kinase inhibitors(TKIs) with or without anti-VEGF; Comparison: TKIs or chemotherapy; Outcomes: OS, PFS; Study design: Randomized controlled trial.**

Condition being studied: **Lung cancer is the leading cause of cancer-related death worldwide. About 85% reported lung cancer cases are non-small-cell lung**

Xie et al. Inplasy protocol 2020100059. doi:10.37766/inplasy2020.10.0059 Downloaded from https://inplasy.com/inplasy-2020-10-0059/

cancer(NSCLC). In the past few decades, epidermal growth factor receptor tyrosine kinase inhibitors(EGFR-TKIs) has improved the clinical prognosis of NSCLC patients, which is definitely the typical example of precision treatment. There are lots of EGFR-TKIs currently in common use, and studies of these drugs have included populations with many different baseline characteristics, including EGFR mutation type. Therefore, we performed this network meta-analysis of randomised controlled trials with latest information to analyze the optimal treatment options for EGFR exon 19 deletion and Leu858Arg mutations patients respectively.

METHODS

Participant or population: Patients with non-small cell lung cancer(NSCLC) harboring epidermal growth factor receptor(EGFR) mutation.

Intervention: Tyrosine kinase inhibitors(TKIs) with or without anti-VEGF.

Comparator: Tyrosine kinase inhibitors(TKIs) or chemotherapy.

Study designs to be included: Any randomized controlled trials(RCTs) involving OS, PFS of TKIs for treating EGFR positive NSCLC will be included.

Eligibility criteria: 1. The searching language is English; 2. All included studies were RCTs and had clinical outcomes, such as OS, PFS; 3. All included studies had clear baseline characteristics of patients and EGFR mutation status; 4. All included studies included subgroup-analysis data required for meta-analysis.

Information sources: Pubmed, Embase, Cochrane Library, ASCO.org, ESMO.org.

Main outcome(s): OS, PFS.

Quality assessment / Risk of bias analysis: We assessed the methodological quality of the included studies by using the Cochrane Collaboratioin's tool for assessing risk of bias. R(ver. 3.6.3) and STATA(ver16.0) were used to assess the quality of studies.

Strategy of data synthesis: We synthesized all direct and indirect evidence to compare the efficacy of different treatments, reported as hazard ratios for survival outcomes (progression free survival and overall survival) along with corresponding 95% confidential intervals. The primary outcome was progression free survival. Secondary outcomes were overall survival. We generated network plots for different outcomes of different targeted patients to illustrate the geometries, to clarify which treatments were compared directly or indirectly in the included studies. We did frequentist, fixed effects, pairwise meta-analysis on head-to-head comparisons based on two or more trials. We assessed heterogeneity between studies using the Q test and I2 statistic within a visual forest plot. Statistical significance was set at a P value of 0.05. Heterogeneity was considered low, moderate, or high for estimated I2 values under 25%, between 25% and 50%, and over 50%, respectively.

Subgroup analysis: We will consider subgroups such as study design.

Sensibility analysis: If significant heterogeneity exists, sensitivity analysis will be performed.

Country(ies) involved: China.

Keywords: NSCLC; TKIs; Network meta-analysis; EGFR mutation type.

Contributions of each author:
Author 1 - Tongji Xie.
Author 2 - Zihua Zou.
Author 3 - Chengcheng Liu.
Author 4 - Yixiang Zhu.
Author 5 - Ziyi Xu.
Author 6 - Le Wang.
Author 7 - Puyuan Xing.
Author 8 - Junling Li.

Xie et al. Inplasy protocol 2020100059. doi:10.37766/inplasy2020.10.0059 Downloaded from https://inplasy.com/inplasy-2020-10-0059/
