# Supplementary material for: Front-Line Therapy in EGFR Exon 19 Deletion and 21 Leu858Arg Mutations in Advanced Non-Small Cell Lung Cancer: A Network Meta-Analysis
Source: Evid Based Complement Alternat Med. 2021 Dec 13;2021:9311875. doi: 10.1155/2021/9311875 (PMC8687779; doi:10.1155/2021/9311875)
Supplement: Supplementary Materials — There are 4 supplementary tables and 2 supplementary figures for this paper. [file 9311875.f1.zip › 9311875.f1/TableS2.docx]

**Table S2. Literature search criteria.**

**Search criteria in Pubmed**

(((((“Carcinoma, Non-Small-Cell Lung”[Mesh]) OR (((((((((non-small-cell lung cancer[Title]) OR (non-small cell lung cancer[Title])) OR (non small-cell lung cancer[Title])) OR (non small cell lung cancer[Title])) OR (non-small-cell lung carcinoma[Title])) OR (non-small cell lung carcinoma[Title])) OR (non small-cell lung carcinoma[Title])) OR (non small cell lung carcinoma[Title])) OR (nsclc[Title])) OR (lung non-squamous[Title])) OR (lung nonsquamous[Title])) OR (lung adenocarcinoma[Title]))) AND ((epidermal growth factor receptor[title/abstract]) OR (EGFR[Title/Abstract]))) AND ((((((((((((((((treatment[Title/Abstract]) OR (therapy[Title/Abstract])) OR (tyrosine kinase inhibitor)) OR (TKI[Title/Abstract])) OR (osimertinib[Title/Abstract])) OR (dacomitinib[Title/Abstract])) OR (afatinib [Title/Abstract])) OR (erlotinib[Title/Abstract])) OR (gefitinib[Title/Abstract])) OR (icotinib[Title/Abstract])) OR (chemotherapy[Title/Abstract])) OR (first-line[Title/Abstract])) OR (first line[Title/Abstract])) OR (treatment-naive[Title/Abstract])) OR (treatment-naïve[Title/Abstract])) OR (untreated[Title/Abstract]))) AND ((((((OS[Title/Abstract]) OR (overall survival[Title/Abstract])) OR (overall-survival[Title/Abstract])) OR (MST[Title/Abstract]) OR (progression-free survival[Title/Abstract]) OR (progression free survival[Title/Abstract]) OR (PFS[Title/Abstract]))) AND (((((((Randomized Controlled Trial[Publication Type]) OR (controlled clinical trial[Publication Type])) OR (randomized[Title/Abstract])) OR (randomised[Title/Abstract]) ) OR (randomly[Title/Abstract])) OR (trial[Title/Abstract])) OR (phase[Title/Abstract])) AND (“0001/01/01”[Date - Publication] : “2020/09/30”[Date - Publication])

**Search criteria in Embase**

('lung tumor'/exp OR 'non-small-cell lung cancer':ti OR 'non-small cell lung cancer':ti OR 'non small-cell lung cancer':ti OR 'non small cell lung cancer':ti OR 'non-small-cell lung carcinoma':ti OR 'non-small cell lung carcinoma':ti OR 'non small-cell lung carcinoma':ti OR 'non small cell lung carcinoma':ti OR 'nsclc':ti OR 'lung non-squamous':ti OR 'lung nonsquamous':ti OR 'lung adenocarcinoma':ti) AND ('epidermal growth factor receptor':ab,ti OR 'EGFR':ab,ti ) AND ('treatment':ab,ti OR 'therapy':ab,ti OR 'tyrosine kinase inhibitor':ab,ti OR 'TKI':ab,ti OR 'osimertinib':ab,ti OR 'dacomitinib':ab,ti OR 'afatinib':ab,ti OR 'erlotinib':ab,ti OR 'gefitinib':ab,ti OR 'icotinib':ab,ti OR 'chemotherapy':ab,ti OR 'first-line':ab,ti OR 'first line':ab,ti OR 'treatment-naive':ab,ti OR 'treatment-naïve':ab,ti OR 'untreated':ab,ti ) AND ('OS':ab,ti OR 'overall survival':ab,ti OR 'overall-survival':ab,ti OR 'MST':ab,ti OR 'progression-free survival':ab,ti OR 'progression free survival':ab,ti OR 'PFS':ab,ti) AND ('Randomized Controlled Trial':it OR 'controlled clinical trial':it OR 'randomized':ab,ti OR 'randomised':ab,ti OR 'randomly':ab,ti OR 'trial':ab,ti OR 'phase':ab,ti) AND [1966-2020]/py

**Search criteria in cochrane**

((non-small-cell lung cancer):ti OR (non-small cell lung cancer):ti OR (non small-cell lung cancer):ti OR (non small cell lung cancer):ti OR (non-small-cell lung carcinoma):ti OR (non-small cell lung carcinoma):ti OR (non small-cell lung carcinoma):ti OR (non small cell lung carcinoma):ti OR (nsclc):ti OR (lung non-squamous):ti OR (lung nonsquamous):ti OR (lung adenocarcinoma):ti) AND ((epidermal growth factor receptor):ti,ab,kw OR (EGFR):ti,ab,kw ) AND ((treatment):ti,ab,kw OR (therapy):ti,ab,kw OR (tyrosine kinase inhibitor):ti,ab,kw OR (TKI):ti,ab,kw OR (osimertinib):ti,ab,kw OR (dacomitinib):ti,ab,kw OR (afatinib):ti,ab,kw OR (erlotinib):ti,ab,kw OR (gefitinib):ti,ab,kw OR (icotinib):ti,ab,kw OR (chemotherapy):ti,ab,kw OR (first-line):ti,ab,kw OR (first line):ti,ab,kw OR (treatment-naive):ti,ab,kw OR (treatment-naïve):ti,ab,kw OR (untreated):ti,ab,kw ) AND ((OS):ti,ab,kw OR (overall survival):ti,ab,kw OR (overall-survival):ti,ab,kw OR (MST):ti,ab,kw OR (progression-free survival):ti,ab,kw OR (progression free survival):ti,ab,kw OR (PFS):ti,ab,kw) AND ((Randomized Controlled Trial):pt OR (controlled clinical trial):pt OR (randomized):ti,ab,kw OR (randomised):ti,ab,kw OR (randomly):ti,ab,kw OR (trial):ti,ab,kw OR (phase):ti,ab,kw)

**Search criteria in ASCO.org and ESMO.org**

NSCLC and EGFR and treatment in 2020
