# Supplementary material for: Front-Line Therapy in EGFR Exon 19 Deletion and 21 Leu858Arg Mutations in Advanced Non-Small Cell Lung Cancer: A Network Meta-Analysis
Source: Evid Based Complement Alternat Med. 2021 Dec 13;2021:9311875. doi: 10.1155/2021/9311875 (PMC8687779; doi:10.1155/2021/9311875)
Supplement: Supplementary Materials — There are 4 supplementary tables and 2 supplementary figures for this paper. [file 9311875.f1.zip › 9311875.f1/TableS3.docx]

**Table S3 Comparisions of the fit of consistency and inconsistency models using deviance information criteria (DIC)**

The DIC is a Bayesian model evaluation criteria that measures model fit adjusted with complexity of the model. Smaller DIC value correspond to more preferable models. Difference between DIC from different models below 5 could be accepted. PFS: progression free survival, OS: overall survival.

Spiegelhalter, D.J., Best, N.G., Carlin, B.P., Van der Linde, A. Bayesian measures of model complexity and fit. Journal of the Royal Statistical Society Series B (Statistical Methodology) 2002; 64(4):583-639

| Model | PFS | | |  | OS | | |
| --- | --- | --- | --- | --- | --- | --- | --- |
|  | All | 19 del | 21L858R |  | All | 19 del | 21L858R |
| Consistency | 66.447 | 36.204 | 33.407 |  | 35.353 | 19.864 | 16.228 |
| Inconsistency | 67.064 | 36.726 | 33.975 |  | 36.201 | 19.461 | 17.648 |
