# Supplementary material for: Front-Line Therapy in EGFR Exon 19 Deletion and 21 Leu858Arg Mutations in Advanced Non-Small Cell Lung Cancer: A Network Meta-Analysis
Source: Evid Based Complement Alternat Med. 2021 Dec 13;2021:9311875. doi: 10.1155/2021/9311875 (PMC8687779; doi:10.1155/2021/9311875)
Supplement: Supplementary Materials — There are 4 supplementary tables and 2 supplementary figures for this paper. [file 9311875.f1.zip › 9311875.f1/TableS4.docx]

**Table S4. Node-splitting analysis of inconsistency.**

All P value was below 0.05, indicating no significant inconsistencies between the direct effect and indirect effects. PFS: progression free survival; OS: overall survival; SoC: standard of care, represents first generation EGFR-TKIs in this network meta-analysis (including gefitinib, erlotinib and icotinib).

| **Nodes** | **Direct effect** | **Indirect effect** | **Overall** | **P** |
| --- | --- | --- | --- | --- |
| **PFS: Overall** |  |  |  |  |
| Chemotherapy, Afatinib | 3.00(2.00-4.30) | 3.30(1.90-5.80) | 3.10(2.30-4.10) | 0.693 |
| SoC, Afatinib | 1.40(0.84-2.20) | 1.20(0.75-1.90) | 1.30(0.92-1.70) | 0.705 |
| SoC, Chemotherapy | 0.41(0.31-0.52) | 0.45(0.25-0.85) | 0.41(0.33-0.52) | 0.720 |
| **PFS: 19 del mutation** |  |  |  |  |
| Chemotherapy, Afatinib | 4.20(2.30-7.70) | 4.30(1.80-11.00) | 4.20(2.70-6.70) | 0.987 |
| SoC, Afatinib | 1.30(0.59-3.00) | 1.30(0.59-2.60) | 1.30(0.77-2.10) | 0.983 |
| SoC, Chemotherapy | 0.30(0.20-0.45) | 0.31(0.11-0.86) | 0.31(0.21-0.43) | 0.966 |
| **PFS: 21L858R mutation** |  |  |  |  |
| Chemotherapy, Afatinib | 2.00(1.00-4.10) | 2.60(0.96-7.30) | 2.20(1.30-3.70) | 0.640 |
| SoC, Afatinib | 1.40(0.55-3.60) | 1.10(0.49-2.60) | 1.20(0.70-2.20) | 0.643 |
| SoC, Chemotherapy | 0.54(0.34-0.83) | 0.70(0.21-2.10) | 0.57(0.38-0.82) | 0.661 |
|  |  |  |  |  |
| **OS: Overall** |  |  |  |  |
| Chemotherapy, Afatinib | 1.20(0.87-1.60) | 1.00(0.64-1.70) | 1.20(0.90-1.50) | 0.603 |
| SoC, Afatinib | 1.20(0.77-1.70) | 1.30(0.88-2.00) | 1.20(0.94-1.70) | 0.617 |
| SoC, Chemotherapy | 1.10(0.84-1.50) | 0.96(0.60-1.60) | 1.10(0.86-1.40) | 0.602 |
| **OS: 19 del mutation** |  |  |  |  |
| Chemotherapy, Afatinib | 1.70(1.10-2.70) | 1.0(0.48-2.20) | 1.50(0.95-2.20) | 0.197 |
| SoC, Afatinib | 1.20(0.64-2.30) | 2.00(1.10-3.70) | 1.60(0.99-2.50) | 0.186 |
| SoC, Chemotherapy | 1.20(0.79-1.80) | 0.70(0.32-1.60) | 1.10(0.73-1.60) | 0.184 |
| **OS: 21L858R mutation** |  |  |  |  |
| Chemotherapy, Afatinib | 0.80(0.51-1.20) | 1.10(0.52-2.20) | 0.87(0.60-1.20) | 0.440 |
| SoC, Afatinib | 1.10(0.62-2.00) | 0.81(0.46-1.50) | 0.95(0.64-1.40) | 0.410 |
| SoC, Chemotherapy | 1.00(0.68-1.50) | 1.40(0.65-2.80) | 1.10(0.78-1.50) | 0.423 |
